# multiSyncPy: A Python package for assessing multivariate coordination dynamics

**DOI:** 10.3758/s13428-022-01855-y

**Published:** 2022-05-05

**Authors:** Dan Hudson, Travis J. Wiltshire, Martin Atzmueller

**Affiliations:** 1 Semantic Information Systems Group, Institute of Computer Science, Osnabrück University, P.O. Box 4469, 49069 Osnabrueck, Germany; 2 Department of Cognitive Science and Artificial Intelligence, Tilburg University, Tilburg, The Netherlands

**Keywords:** Synchrony, Coordination, Dynamics, Interaction, Multivariate methods

## Abstract

In order to support the burgeoning field of research into intra- and interpersonal synchrony, we present an open-source software package: multiSyncPy. Multivariate synchrony goes beyond the bivariate case and can be useful for quantifying how groups, teams, and families coordinate their behaviors, or estimating the degree to which multiple modalities from an individual become synchronized. Our package includes state-of-the-art multivariate methods including symbolic entropy, multidimensional recurrence quantification analysis, coherence (with an additional sum-normalized modification), the cluster-phase ‘Rho’ metric, and a statistical test based on the Kuramoto order parameter. We also include functions for two surrogation techniques to compare the observed coordination dynamics with chance levels and a windowing function to examine time-varying coordination for most of the measures. Taken together, our collation and presentation of these methods make the study of interpersonal synchronization and coordination dynamics applicable to larger, more complex and often more ecologically valid study designs. In this work, we summarize the relevant theoretical background and present illustrative practical examples, lessons learned, as well as guidance for the usage of our package – using synthetic as well as empirical data. Furthermore, we provide a discussion of our work and software and outline interesting further directions and perspectives. multiSyncPy is freely available under the LGPL license at: *https://github.com/cslab-hub/multiSyncPy*, and also available at the Python package index.

## Introduction

Across physical, biological, and social systems, interacting components of complex systems coordinate and, at times, synchronize their behavior. When two or more system components are aligned temporally and spatially, then their behavior is thought to be synchronized. Synchronization is a well-known natural phenomenon, and a seemingly universal property exhibited by complex systems (Amazeen, 2018; Kelso, 1995; Lee, 2005; Pikovsky et al., 2001; Xuan et al., 2018; Xuan & Filkov, 2013) appearing in the temporal alignment, for example, in the cycles of subpopulations of cells (Banfalvi, 2017), in the oscillations of pendulums and brainwaves (Dikker et al., 2017; Lai et al., 2006), light pulses in groups of fireflies (Strogatz & Stewart, 1993), primate interaction behaviors (Yu & Tomonaga, 2015), physical networks such as power grids (Motter et al., 2013), and in a variety of physiological and behavioral modalities of interacting humans (Feldman, 2007; Hoehl et al., 2020; Palumbo et al., 2017; Wiltshire, Philipsen, et al., 2020a). Despite its pervasiveness, there is still much uncertainty about how synchronization originates in systems, its functional role in different contexts and different modalities, what forms it takes in groups larger than two (or between more than two variables), and how it changes over time (Duranton & Gaunet, 2016; Hoehl et al., 2020; Knoblich et al., 2011; Launay et al., 2016; Mayo & Gordon, 2020; Nowak et al., 2017; Timmons et al., 2015; Wiltshire, Steffensen, et al., 2020b).

In order to advance the scientific exploration of multivariate *coordination dynamics*, an area of inquiry that “describes, explains and predicts how patterns of coordination form, adapt, persist and change in living things” (p. 1537, Kelso, 2009), this paper, and the corresponding Python package, focuses on metrics that are used to measure multivariate synchrony. *Coordination,* in the broadest sense, is a behavior exhibited by dynamical systems that is sometimes recognized as a superordinate construct characterizing the ways in which components and processes of complex dynamical systems covary over time (Butner et al., 2014; Richardson et al., 2013; Turvey, 1990; Wiltshire, Philipsen, et al., 2020a), in which phenomena like synchronization, coupling, alignment, entrainment, behavior matching, and so on are *forms* of coordination (Butler, 2011). While some work aims to differentiate these forms of coordination, in our work, we use these terms somewhat interchangeably with the consistent aspect being that they are all concerned with the temporal covariation of multivariate time series.

While there has been much work on the bivariate and dyadic synchronization (Abney et al., 2015; Altmann et al., 2020; Cohen et al., 2021; Delaherche et al., 2012; Likens & Wiltshire, 2020; Louwerse et al., 2012; Palumbo et al., 2017; Paulick et al., 2018; Ramseyer & Tschacher, 2011; Tschacher & Meier, 2020; Wiltshire, Steffensen, et al., 2020b), there are relatively fewer cases examining multivariate synchrony (e.g., Butner et al., 2014; Dias et al., 2019; Reinero et al., 2020; Wallot & Leonardi, 2018; Zhang et al., 2019), which can be useful for understanding how for example groups, teams, and families coordinate their behaviors, or how multiple modalities from an individual can become synchronized. While the relatively simpler bivariate case only considers dyadic (pairwise) relationships, multivariate synchrony, in general, extends this beyond dyads such that, e.g., triads or even larger groups can be analyzed.

Past work on interpersonal coordination comprises a diverse body of work with investigations in general social interactions (Abney et al., 2015; Chanel et al., 2013; Louwerse et al., 2012), parent–child interactions (Abney et al., 2017; Crowell et al., 2017; Feldman, 2007; Nguyen et al., 2020), child–child interactions (Altmann, 2011), romantic partners (Butler & Barnard, 2019; Gottman, 2014; Randall et al., 2013; Timmons et al., 2015), families (Butner et al., 2018), strangers and friends (Bizzego et al., 2020; Galati et al., 2020), mental health-related interactions (Butner et al., 2017; Ramseyer & Tschacher, 2011; Soma et al., 2019; Wiltshire, Philipsen, et al., 2020), teamwork (Dias et al., 2019; Likens et al., 2014; Palumbo et al., 2017; Reinero et al., 2020; Wiltshire et al., 2019), performance groups (Keller et al., 2014; Setzler & Goldstone, 2020), and even inter-species interactions (Wanser et al., 2021). In addition, there is evidence of synchronization phenomena in many modalities including non-verbal behaviors and movements (Ramseyer, 2019; Schoenherr et al., 2019), acoustic properties of speech (Fischer et al., 2017; Imel et al., 2014; Wieder & Wiltshire, 2020), alignment and matching in language (Duran et al., 2019; Fusaroli & Tylén, 2016; Lord et al., 2015; Niederhoffer & Pennebaker, 2002), physiological signals from the autonomic nervous system (Kleinbub, 2017; Kleinbub et al., 2020; Konvalinka et al., 2011), patterns of neural activation (Dikker et al., 2017; Hoehl et al., 2020; Koban et al., 2019), and between multiple modalities (Amon et al., 2019; Gorman et al., 2016).

Not only this, but a growing number of options exist for measuring signals to examine interpersonal coordination, ranging from the traditional method of hand coding video frames (Bernieri & Rosenthal, 1991), motion capture systems (Romero et al., 2017), video and audio and speech processing (Cao et al., 2021; Kleinbub & Ramseyer, 2020; Paxton & Dale, 2013; Pouw et al., 2020; Vilela Barbosa et al., 2012; Weusthoff et al., 2018), physiological sensors (Guastello & Peressini, 2017; Palumbo et al., 2017), neuroimaging devices (Dumas et al., 2010, 2011; Reindl et al., 2018), and sociometric sensors (Kozlowski, 2015; Montanari et al., 2018; Parker et al., 2018). To be able to measure coordination in a variety of modalities and across groups or teams of sizes three or greater, different methods are needed. It has been toward that aim that we developed this package and in which we include a measure based on Symbolic Entropy, Multidimensional Recurrence Quantification Analysis (mdRQA), Coherence (and a related but newly proposed ‘Sum-Normalized cross-spectral density (CSD)’), the Cluster-Phase ‘Rho’ metric, and a statistical test based on the Kuramoto order parameter. That being said, while many of the metrics we present have been utilized for examining the coordination of behavioral and physiological signals in these inherently social contexts, the methods are also applicable to other pertinent research contexts such as human–computer interaction (Novick & Gris, 2014) that look at coordination properties of a variety of multivariate signal streams.

In this paper, we present multiSyncPy, a Python package for computing a variety of synchrony metrics on multivariate time series. Our aim in developing this package is to make these methods more accessible and to encourage their systematic use to enrich our understanding of coordination dynamics beyond the dyad (Amon et al., 2019). In the next section, we discuss related past work, focusing on the background of the metrics included in our Python package while highlighting important considerations for their use in research. After that, we present the contents of the package and show how to use it with some example code. Following this, we demonstrate the use of our multivariate synchrony metrics on a series of synthetic datasets and two real-world empirical datasets, showcasing the results obtained from a variety of situations.

Importantly, we aim to explore the performance of the multivariate synchrony metrics included in multiSyncPy on a variety of datasets and types as there are always a number of decisions to be made. Thus, we also provide several lessons learned from this initial investigation. In particular, using synthetic data from autoregressive processes with additional correlated noise, we find that some metrics respond more strongly than others to unstructured noise that is duplicated across all variables. Synthetic data from Kuramoto models is used to show that the metrics are capable of distinguishing different degrees of coupling; it also provides evidence of convergent validity between different multivariate synchrony metrics as well as an aggregated version of a well-established bivariate synchrony metric. Two empirical datasets provide realistic examples of how multiSyncPy can be used, specifically when investigating synchronization in body movement data from groups with more than two members. Finally, in our paper, we conclude with a summary, discuss our work in its broader scientific context, and highlight some important future directions.

## Multivariate synchronization metrics in multiSyncPy

### Symbolic entropy

One approach to the investigation of synchronization in multi-component systems is investigating the level of temporal regularity of state sequences. Since complex systems have many components (Amazeen, 2018; Favela, 2020), they exhibit a large number of possible states and can cycle through varied combinations of these behavioral states. If the components of the system are synchronized, then the system will exhibit a smaller number of different states and it will do so with increased repetitiveness. One approach in this vein is what we will refer to as ‘symbolic entropy’. This information-theoretic approach, following the work of Stevens and colleagues on neurodynamic and physiological synchronization in teams (Dias et al., 2019; Likens et al., 2014; Stevens, 2012; Stevens et al., 2012; Stevens & Galloway, 2014), aims to characterize the state of the system at each point in time as a discrete value, and examines the entropy of these discrete states over some window of time. Low values of entropy are considered indicative of behavior synchronized around some shared, regular and ordered pattern. This method can readily apply to nominal data, but additionally, to obtain discrete states from continuous measurements, each variable can be individually mapped to a value of either ‘low’, ‘medium,’ or ‘high’ at each time step (or some other discretization procedure). Each conjunction of low, medium, and high values in the variables in the system then becomes an element in a symbol set that characterizes the overall collective system state for a given observation, where for example, a symbol for a three-person team could be one that captures the high, high, low pattern and so on (Dias et al., 2019; Stevens et al., 2019).

Each method for measuring synchronization has its advantages and disadvantages, which often relate to the type of data being analyzed. In the case of symbolic entropy, one consideration is that the number of possible system states increases exponentially with the number of components in the system, potentially to the point that entropy becomes hard to estimate without an extremely long time series to analyze. Since entropy is based on an estimation of the probability of the different states occurring, with many states, a long time series may be required in order for all possible states of the system to be observed enough times to reliably estimate their probabilities. Additionally, because entropy is affected by the number of possible states, symbolic entropy scores should be compared between time series with the same number of variables. An advantage of the entropy-based method is that it makes no assumptions about the temporal signature so it can apply to cyclical or non-cyclical signals. And, even if it is difficult to get a reliable estimate of the state probabilities, it is still suitable for relative synchrony comparisons between, for example, experimental conditions assuming the lengths of time series are approximately equal.

Another consideration is that our implementation of symbolic entropy is based on categorizing values into ‘low’, ‘medium’, and ‘high’ based on tercile boundaries. Components that are synchronized at a high frequency might pass through the tercile boundaries (and so have increased entropy) compared to unrelated components that each have a low frequency. Note that other methods do exist for generating symbolic states from continuous time series (Cysarz et al., 2013; Qumar et al., 2013). Finally, it is important to remember that low entropy is not the same as synchronization, and this could reflect other phenomena, such as a period of rest in which the components of the system stay at ‘low’ measurement values for some amount of time. Some options like surrogate testing, which will be discussed later, might help to distinguish synchrony from other phenomena leading to low entropy.

### mdRQA

Similar to symbolic entropy, multidimensional recurrence quantification analysis (mdRQA) uses the temporal regularity and recurrence of system states and sequences of states as a proxy for synchronization (Wallot & Leonardi, 2018). The regularity of the system is described using a binary recurrence matrix that indicates which points in time are similar to which other points in time (Coco et al., 2020). For a given state to count as recurrent, the similarity of two states is typically determined using the Euclidean distance, and then a radius threshold is applied to provide a binary classification: states are either ‘recurrent’, meaning that they are sufficiently similar, or not. The results of these binary classifications form a square matrix, where the row index and the column index both specify times (the times being compared). Multivariate time series data series can either be entered ‘raw’ into mdRQA or following time-delayed embedding to reconstruct higher-dimensional dynamics of the system (Takens, 1981; Wallot & Leonardi, 2018). Recent methods for selecting appropriate multidimensional delay and embedding dimension parameters have also been developed (Wallot & Mønster, 2018).

Once a recurrence matrix has been created, mdRQA proceeds by computing metrics that summarize the recurrence observed in the system. These are based on the diagonals of the recurrence matrix, which represent the initial time series compared to itself at some particular time delay. For example, the main diagonal represents a comparison of the system to itself without any time delay, and so is always populated entirely with ones – indicating that the system is recurrent, or similar to itself, all the way along. Diagonals close to the main diagonal represent a comparison of the system to itself with a short delay, while diagonals further from the main diagonal represent a comparison with a longer delay. Four main metrics are commonly used to summarize the recurrence matrix. All of these metrics consider only cells that are off the main diagonal, which is an assumption of the following descriptions. The proportion of recurrence (%REC) is simply the number of recurrent cells divided by the total number of cells. The proportion of determinism (%DET) only considers sequences of diagonally recurrent cells that are longer than a specified length. It is the number of recurrent cells left after applying this criterion, divided by the number of recurrent cells. The average length of a diagonal sequence (ADL) of recurrent cells provides a third metric, and the length of the longest diagonal sequence provides the final metric (maxL).

One of the benefits of recurrence-based analysis is that it is considered to handle nonlinearity and nonstationarity well, since it does not explicitly model the variables or their interactions as some particular set of functions (Wallot & Leonardi, 2018; Webber & Marwan, 2015; Webber & Zbilut, 2005). This may make mdRQA more desirable than other metrics in contexts where nonlinearity and nonstationarity are expected. If mdRQA is chosen, there are some additional considerations that do not apply to other metrics for synchrony. First, there is the ‘radius’ parameter, which cannot typically be decided analytically, and so an appropriate value must be determined empirically by iteratively running the analysis with different values until a reasonable amount of recurrence is returned, typically between 1 and 5% recurrence (for further discussion and insights on setting the radius and other mdRQA parameters see Wallot & Leonardi, 2018; Wallot & Mønster, 2018). Because an unweighted Euclidean distance is used to compare time steps, normalization is also an important part of data processing, to make sure that all variables vary across a similar scale. A final point to note is that, like with symbolic entropy, the recurrence in a system is not exactly the same as synchrony and the difference might be noticeable in the results when looking at periods of relatively low activity (which could have high recurrence though little synchronization).

### Coherence

Another method used to measure group synchrony is averaging of spectral coherence scores. This is based on examining the power spectrum of each variable through spectral decomposition and comparing the power at each frequency between signals (White, 1984). Mathematical details and sensitivity analysis for this measure can be found in (Winterhalder et al., 2006). Ultimately, the metric ranges from 0 to 1 indicating how well one signal can be approximated by a linear function of the other signal. As described so far, the spectral coherence metric only operates with two signals. However, recent work by Reinero et al. (2021) has used a multivariate version of coherence in the context of comparing synchrony between individuals in EEG recordings across multiple frequency bands (Reinero et al., 2021). Their method provided aggregated scores across a team by simply averaging across frequencies and across participants. This process is what we use in our software package to offer a multivariate synchrony metric based on coherence.

There are two important assumptions to the spectral coherence measure of synchrony: that the signals are related by a linear rather than nonlinear function, and that the signals are stationary (White & Boashash, 1990). In contexts where these assumptions are violated, this may mean that the performance of the synchrony metric is impaired. Another note about this metric is that it relies on cross-spectral density, which may be difficult to reliably compute with lower sampling frequencies or shorter time series.

There is one additional and important consideration for the averaged coherence metric. This method uses an average across frequencies of the coherence, which is a normalized value (ranging between 0 and 1). This means that information about the relative amplitude at different frequencies is ignored, which may be undesirable for some types of signals. For example, this issue often becomes noticeable when a recording includes Gaussian noise, since Gaussian noise impacts the spectral content at all frequencies, whilst the meaningful content of the ‘true’ signal may only be contained in a limited range of frequencies. If this is the case, then the Gaussian noise may dominate over the meaningful content when averaging across frequencies, especially when using a high sampling rate which allows for many frequency components to be computed. If possible, the reliability of the metric could be improved if filters are applied to remove noise such as a bandpass filter that removes noise occurring at irrelevant frequencies.

If filtering is not possible, noise may have a serious impact on the results. To mitigate this issue, we propose an additional metric that is closely related to the averaged spectral coherence. Observing that the coherence value at each frequency component is a normalized version of the cross-spectral density (CSD) at that component, we propose to use the cross-spectral density to define an additional metric, but postpone normalization until after aggregating the values across frequencies. As a consequence, information contained in the cross-spectral density regarding amplitude can be used to moderate the impact that each frequency component has on the final output. Our proposal is to use the sum across frequencies of the squared cross-spectral density, and normalize by the sum across frequencies of the auto-spectral density of the first signal multiplied by the auto-spectral density of the second signal. This then produces a value between 0 and 1 for a pair of variables. For two variables, the calculation is as follows:

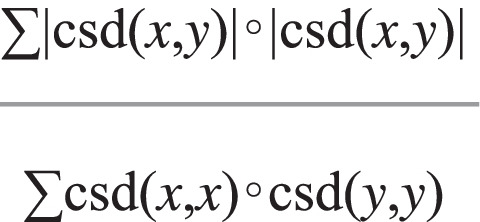



where each sum is across *n* frequency components. Repeating the process across all pairs of variables and then averaging leads to a final multivariate metric. Hereafter, we shall refer to this additional metric as “sum-normalized CSD”. This metric may be preferable when there is substantial noise that cannot be filtered out, for example, because it is in a frequency range of interest.

A final point to note about both the aggregated coherence and sum-normalized CSD is that they are based on estimating the spectral density at different frequencies. As with any time-frequency analysis, the number of frequencies that can be analyzed varies according to the length of the input time series, making it difficult to compare results from multivariate time series of different lengths.

### Rho

The remaining two synchrony metrics are based on the concept of the phase of a periodic signal, which describes how far the signal is along its cycle of behavior, at any given moment in time (Haken et al., 1985). Signals can be compared based on how similar their phases are over time using, for example, the relative phase measure (Lamb & Stöckl, 2014), which is the difference in phase between signals.

Richardson et al. (2012) developed a ‘cluster-phase’ method, which looks at an aggregate relative phase across multiple signals, and then computes how closely the phases of each individual signal are to the aggregate-level phase. Their method is able to provide an overall measure of synchronization across an entire set of signals, and also makes it possible to obtain a synchrony estimate at each point in time.

Before these analyses can be completed, it is necessary to extract phase information from the raw signals. There are various ways of doing this, which take a time series of amplitude measurements as an input and return a time series of phase values as an output. Two of the most common methods are (1) to perform the Hilbert transform and then calculate angles from the resulting complex numbers, and (2) to perform wavelet analysis (Issartel et al., 2015). This is a necessary step in data preparation that the analyst must decide. One potential issue for the cluster-phase rho metric is that it may be more difficult to extract reliable phase information from quasi-periodic signals (see Hurtado et al., 2004 for some strategies to mitigate this). Extracting meaningful phase information is a precondition for obtaining meaningful results from the ‘rho’ metric.

### Kuramoto weak null test

The next metric operates on a collection of several multivariate recordings, rather than a single recording, and provides an estimation of the statistical significance of the synchrony observed in the collection, based on a null hypothesis that the observed levels of synchrony are due to chance. The method is based on the relative phases of the multiple variables in each recording, which are summarized using the Kuramoto order parameter. This ‘order parameter’ is based on Kuramoto’s mathematical model of coupled oscillators, and represents a key value for describing the behavior in the model (Kuramoto, 1975; O’Keeffe et al., 2017). The values observed for the order parameter across the sample can then be analyzed with reference to the values that would be expected due to chance, leading to a statistical test for significant levels of synchrony in the sample. The Kuramoto order parameter is based on the idea that if a system is composed of oscillators that are coupled to each other with equal strength, then the oscillators will experience some attraction to the average phase (the average across all oscillators in the system). There are some free parameters for the model that will determine what proportion of the oscillators in the system will synchronize. First, there is the *coupling strength*, which represents how strongly each oscillator influences the others. Second, each oscillator has its own *natural frequency*, and an oscillator’s preference for its natural frequency may pull it away from a shared/common frequency of synchronization. This mathematical model of course relies upon some simplifying assumptions, specifically that the oscillators follow sinusoidal patterns of amplitude and that they are all equally coupled to each other, which may not be true in all real-world examples. Nevertheless, it is a highly influential model of synchronization and is often used to model real-world data.

Frank and Richardson (2010) constructed null hypotheses for the Kuramoto order parameter values observed across a sample of recordings. These hypotheses predict a probability distribution of what will be observed when sampling oscillators that are not coupled to one another. We focus on the ‘weak null hypothesis’ and associated test for significance, which does not assume that autocorrelation is absent in the variables, since this is more general than the alternative ‘strong null’, which does make that assumption. The ‘weak null’ was deemed more appropriate because it relies on fewer assumptions and so is applicable to a wider range of scenarios (Frank & Richardson, 2010). Before running the test, we recommend visually inspecting the distribution of phase values that were extracted during data preparation from the variables in the time series of the sample, because it is important to check the assumption that each variable has an approximately uniform distribution of phase values. An uneven distribution of extracted phase values can lead to highly overestimated significance.

## Overview and examples of the multiSyncPy package

A number of packages in different programming languages exist to calculate synchronization measures. Gouhier and Guichard (2014) presented one such package, named ‘synchrony’, for the R statistical programming language, offering Kendall’s W, Loreau and de Mazancourt’s φ and two ‘nonlinear’ metrics based on similarities between phase as determined after applying a preliminary peak-picking step, as synchrony metrics to use on multivariate time series. Wallot and Leonardi (2018) created functions for the R programming language for performing multidimensional recurrence quantification analysis (‘mdRQA’) on multivariate time series, and the same functionality was also presented by Wallot et al. (2016) in the context of MATLAB instead of R. For the Python programming language, there is the syncPy package from Varni et al. (2015) for analyzing synchrony in dyadic or multiparty contexts, which mostly provides metrics designed for bivariate time series, although some methods work for multivariate data, specifically: Granger causality, Omega complexity, the S-Estimator, and partial coherence. The scientific analysis package Scipy (Virtanen et al., 2020) contains digital signal processing methods such as spectral coherence, which can be used to quantify synchronization, although these are limited to bivariate data. For the analysis of neuronal activity, Mulansky and Kreuz (2016) introduce PySpike, which provides various methods for computing spike chain synchrony. Functions for quantifying synchrony are also available for MATLAB, such as in the HERMES toolbox of Niso et al. (2013), which primarily focuses on bivariate synchrony, but does include the multivariate ‘synchronization likelihood’ metric. Recently, Baboukani et al. (2019) created a MATLAB package that provides the ability to calculate four different multivariate synchronization metrics. These tools are published openly, but they are tied to MATLAB, which requires a paid license.

Our Python package is unique in that it is the only package we know that is completely free to use that focuses exclusively on multivariate synchrony, and the only one to offer our particular combination of metrics, the value of which is demonstrated by the fact that these metrics have been used in recent empirical investigations of synchronization. As previously stated and reviewed, six types of multivariate synchrony analysis are included in our package: symbolic entropy, multidimensional recurrence quantification analysis (mdRQA), coherence, the cluster-phase ‘rho’ metric, and a statistical test based on the Kuramoto order parameter (based on Frank and Richardson’s weak null hypothesis), plus our proposed ‘sum-normalized CSD’.

In addition to the different multivariate synchronization methods mentioned above, multiSyncPy also offers functions to generate surrogate datasets from samples containing several multivariate time series. Surrogate data is often used in synchronization-focused research as a way to determine whether or not the observed dynamics are different than chance levels (Moulder et al., 2018; Schreiber & Schmitz, 1996; Strang et al., 2014; Theiler et al., 1992). This is useful because in many cases it is not possible to deduce the likelihood of observed synchrony scores without an appropriate null hypothesis value to compare to. We offer two ways to create surrogate data: segment shuffling and variable swapping. Segment shuffling first involves dividing each variable in a time series into windows, and shuffling the windows independently for each variable. This aims to preserve most of the structure of the signals while removing temporal relationships arising from synchronization. The second method is to swap variables across time series, by separating out the variables and then rearranging into new time series, so that temporal relationships between observations are lost, but each signal and its temporal relationships with itself are retained. For example, if there are three time series consisting of variables (X,Y,Z), (U,V,W) and (R,S,T), then after swapping variables the surrogate time series might be (X,U,R), (Y,V,S) and (Z,W,T). This requires that the time series have the same number of variables and time steps, but is desirable because it preserves the full structure of the signals.

**Fig. 1:**
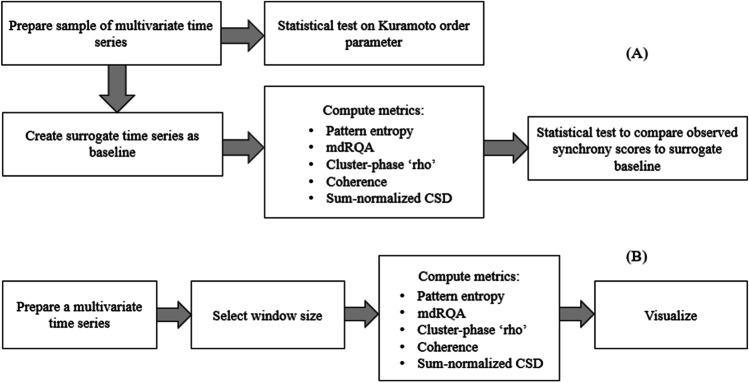
Example workflows for analysis with multiSyncPy

Prior to giving our demonstration of how to compute the different metrics multiSyncPy offers using Python, we give an overview of the workflow and then details of the synthetic data we generated. As a *scientist* prepared to analyze data from an experiment, in which they wish to establish synchronization within a collection of multivariate recordings, the typical workflow is shown below in Fig. 1A. First, the scientist would prepare the sample of time series that they want to analyze by doing any necessary pre-processing, for example by removing outliers or applying a bandpass filter to remove noise from the recordings, and by preparing corresponding phase time series to be used with the phase-based metrics. If the variables in a multivariate time series are of different lengths (e.g., R-R intervals from a group), then time normalization may be necessary. Testing the Kuramoto order parameter can be done directly on the prepared time series, while surrogate data should be prepared in order to test the other metrics. When using surrogate data, metrics should be calculated on both the prepared sample and the surrogate sample, and the resultant values can then be compared via a *t* test. In the case that two experimental conditions are being compared, it may be preferable to simplify the workflow by not creating surrogate data or performing the test on the Kuramoto order parameter, and instead comparing the computed synchrony metrics between the two conditions.

Another workflow could be more *practitioner-oriented*, suited for those studying groups or teams and aiming to provide immediate feedback on their coordination. This workflow is shown in Fig. 1B. The data should be prepared with any necessary pre-processing, for example by removing outliers or applying filtering, and by preparing corresponding phase time series. A window size should be chosen within which to calculate the various metrics. The metrics can then be computed and their progression over time can be visualized as a time series plot, which can then be presented by the practitioner for interpretation and discussion. The multiSyncPy package includes a windowing function that allows the user to estimate changes in synchrony over time for most of the measures. 

In addition to offering the synchrony metrics and surrogation methods mentioned previously, multiSyncPy also provides the ability to generate two types of synthetic time series to support users’ initial exploration of the synchrony metrics. In fact, we make use of this synthetic data generation to showcase our code, and we also use it in the subsequent section to test out the results given by each metric, by systematically varying the properties of the synthetic data.

The first kind of synthetic data is generated using a stochastic autoregressive function (Gouhier & Guichard, 2014). At each time step, this computes the new value based on a weighted sum of the previous two values and some additional Gaussian noise. Iterating the process enough times will lead to a time series of any desired length. This is used to produce multiple univariate times series of the same length, which can then be stacked alongside one another and treated as the different variables of a multivariate time series. It is important to note that, due to the stochastic nature of the autoregressive function used, and the fact that the univariate time series are created independently, there is no coupling or other interaction between the variables that would lead to synchrony. The different variables do however exhibit a similar autocorrelation since they come from the same class of process. This synthetic data therefore serves as our ‘null scenario’ in which the amount of synchrony computed should not be above chance level.

The second process used to generate synthetic data is a Kuramoto model (Kuramoto, 1975), which models the behavior of coupled oscillators over time. Each oscillator has its own natural frequency at which it cycles when there is no influence from the others, and the model overall has a coupling strength parameter which reflects how strongly each oscillator influences the others. Our implementation also adds a small amount of Gaussian noise to make the data more naturalistic. Keeping the natural frequencies of the oscillators the same whilst increasing the coupling strength should lead to higher levels of synchrony. We investigate the extent to which the synchrony metrics match to this expectation. The outputs of the Kuramoto model are naturally multivariate, with a variable corresponding to each simulated oscillator. The model specifies how to update the phase of each oscillator at each time step, from the phases at the previous time step. Iterating this procedure leads to a time series of any desired length. In contrast to the autoregressive data, which produces variables independently with no above chance-level synchrony expected, the data from the Kuramoto model is expected to exhibit synchrony as the coupling strength is increased. Synthetic data from the Kuramoto model therefore constitutes a verifiable example of multivariate synchrony, which should be reflected in the values computed for our synchrony metrics.

### Computing the synchronization metrics

First of all, before computing the synchronization metrics, we import multiSyncPy and related packages into Python, as below.

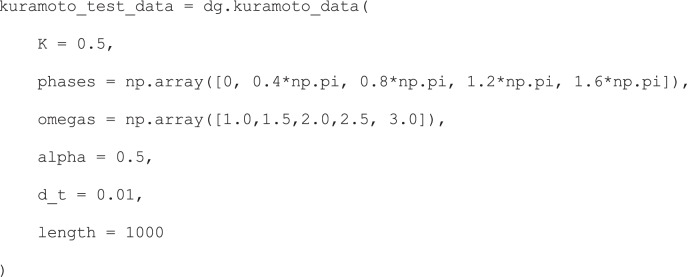



Next, for the purposes of illustration, we generate some synthetic data on which to compute synchrony metrics, in this case using the Kuramoto model. The function ‘kuramoto_data’ requires specification of multiple parameters, which correspond to the parameters of the Kuramoto model (described in more detail in the next section). The parameter K is the coupling strength. The initial phases for the oscillators are provided as a numpy array, and so too are the natural frequencies in the parameter ‘omegas’. The alpha value modulates the contribution of Gaussian noise to the signals. The standard deviation of the Gaussian noise is the square root of parameter ‘d_t’, which is the length in seconds of the period between time steps, and the noise is multiplied by alpha before being added. The length is the number of time steps to generate. Below is the code to generate some example data.

 The function returns a numpy array of shape (number_oscillators, sequence_length). Note that the numpy array is the data structure used across our package to represent multivariate time series.

If we had chosen to use autoregressive synthetic data instead of data from a Kuramoto model to showcase our code, the synthetic data would be generated using the following code. The length must be specified, along with ‘phi_1’ and ‘phi_2’, respectively, the weighting of the values one and two time steps ago in the autoregressive process, ‘epsilon’ which is the standard deviation of Gaussian noise added at each time step, and an optional bias term ‘c’. These parameters correspond to the parameters of the autoregressive process described in more detail in the next section.



This returns a univariate time series of the desired length. To construct a multivariate time series, multiple univariate time series would be generated and stacked together.

With some data on which to compute the metrics, now it is possible to calculate the symbolic entropy. This requires only a simple function call:

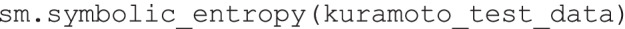



The symbolic entropy across the entire time series is returned as a single number.

For mdRQA, our package includes a function to create the recurrence matrix for a multivariate time series. The user must specify a ‘radius’, which is used as a threshold to decide when two time points are sufficiently similar to be considered recurrent. If an appropriate value is not known a priori, then typically the radius is established by iteratively running the mdRQA analysis and adjusting the radius until the percentage of recurrence is between 1 and 5 (see Wallot & Leonardi, 2018). By default, normalization is applied so that each variable has a mean of 0 and variance of 1 before computing Euclidean distances and deciding which points count as recurrent. In addition, users can also optionally provide an ‘embedding dimension’ and a corresponding delay parameter to use when constructing the recurrence matrix, if they want to use embedding in the mdRQA. Using embedding is not obligatory however, and its value and validity when applied to multivariate time series is still open to discussion. Wallot and Leonardi (2018) provide a more detailed explanation of embedding in recurrence quantification analysis and a discussion about its use with multivariate time series. With the recurrence matrix available, the mdRQA metrics can be computed easily:

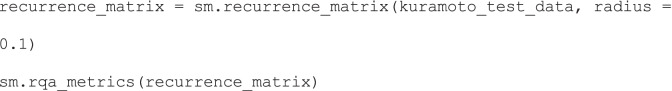



The ‘rqa_metrics’ function returns four values in a tuple: proportion of recurrence (%REC), proportion of determinism (%DET), mean diagonal length (ADL), and max diagonal length (maxL). Each of these are a single number.

The next metric is the aggregated coherence score, as used in Reinero et al. (2021). Once again, this is a simple function call on a numpy array containing our multivariate data. The coherence is returned as a single number which is the aggregation of the pairwise coherence values. The code is as follows:



It is worth noting that the aggregated coherence score can be affected by the presence of Gaussian noise, and if this is likely, then it may be valuable to also compute the ‘sum-normalized CSD’ metric proposed in this paper, as follows:


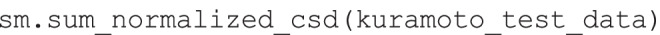


The remaining metrics are slightly different because they are based upon analyzing the phase time series of the different variables. This means that it is necessary to convert the raw amplitude values in the time series into phase values. Because there are multiple valid ways to do so, such as extracting them from the Hilbert transform or using wavelet analysis, it is left open to the user to decide which method to use. The example code below shows how to use functions from the numpy (van der Walt et al., 2011) and scipy (Virtanen et al., 2020) packages to obtain phase time series via the Hilbert transform (including a refinement to normalize the data to have a mean value of zero, which facilitates the phase calculation):



Given estimation of the phase angle time series, it is now possible to compute the cluster-phase ‘rho’ metric described by Richardson et al. (2012), and here it can be called using the ‘rho’ function as follows:

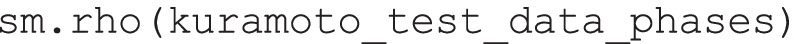



The cluster-phase ‘rho’ metric is different from those showcased above, in that it provides both a time-varying synchrony estimate as well as an overall estimate for the entire time series. For this reason, the rho function returns two objects: (1) a numpy array of length equal to the length of the input time series, which is a continuous estimate of synchrony at each moment, and (2) the overall score as a single value.

By default our ‘rho’ function returns a continuous and time-varying estimate of synchronization. In contrast, we also provide a windowing function that allows users to do the same in order examine the development of synchrony over time using the other metrics. In other words, by default a majority of these metrics return values that summarize the entire time series, but our windowing function allows one to conveniently estimate the change in coordination over time. The user simply provides the time series data, the function used to compute a specific metric, the number of time steps to use as a window, and the number of time steps to use as a step size between successive windows. The outputs are provided in a numpy array with the first dimension representing windows, and the other dimensions being determined by the synchrony metric in question. For example, to calculate the symbolic entropy in windows of size 100 with an offset of 100 and thus, no overlap, the code is as follows:

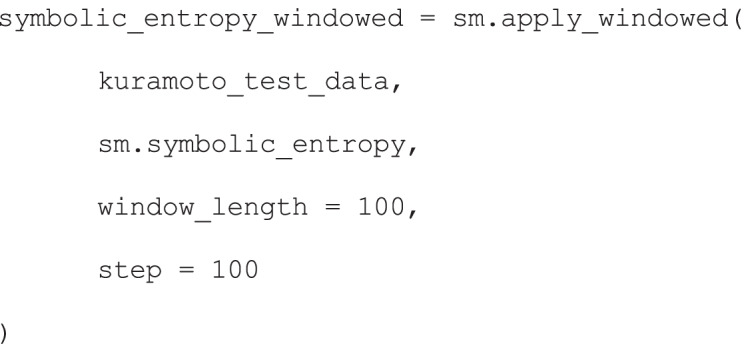



The final synchrony metric included in our package is a statistical test on the Kuramoto order parameter, to determine how likely a sample of data is according to the weak null hypothesis of Frank and Richardson (2010). Unlike the methods described previously, the statistical test operates over a sample of data, and so multiple time series are required. The Kuramoto order parameter values of multiple different time series, for example coming from multiple runs of an experiment, are put into an aggregate comparison to the values expected under a null hypothesis that synchronization is not occurring. Therefore, the function to compute this metric requires as input a list of numpy arrays which each have the shape (number_variables, length_of_time_series). The lengths of the multivariate time series can vary (as long as each variable in a particular multivariate time series is the same length), but they must all have the same number of variables since this affects the predicted values under the null hypothesis. Moreover, it is important to remember that *the inputs should be the phase time series rather than the raw amplitude values*. To demonstrate this function, we generate a sample of multivariate time series from Kuramoto models and convert them into phase time series.

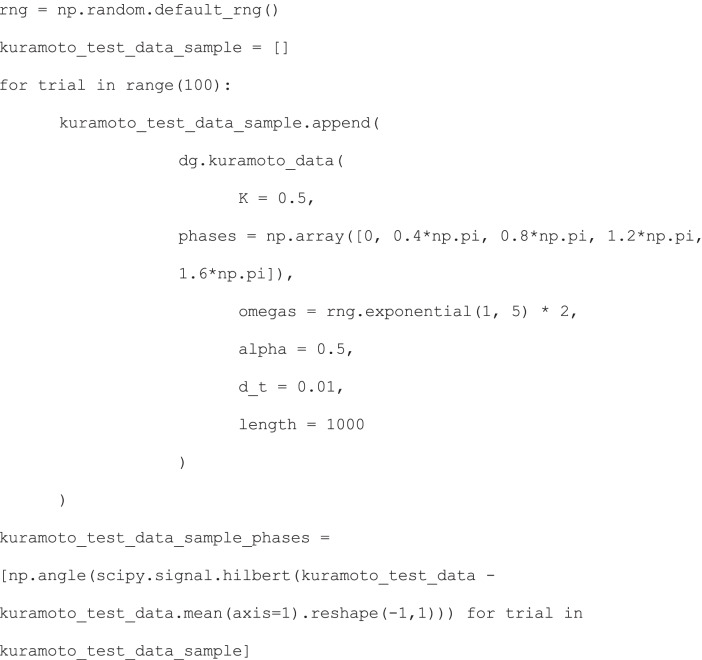



Once the synthetic data are available, performing the test can be done using the ‘kuramoto_weak_null’ function as follows:

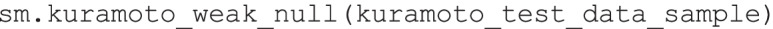



This function returns the *p* value, *t*-statistic, and degrees of freedom for the sample provided.

Lastly, multiSyncPy provides two functions for generating surrogate data from a sample of time series, which for example can then be used to calculate baseline results. The first cuts each variable in each time series of the sample into windows, and reorders the windows. The code to construct this type of surrogate data from a sample is as follows. A list containing numpy arrays of shape (number_variables, number_time_steps) is required, as is the desired length of a window.

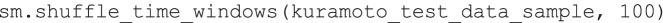



The second method for constructing surrogate data works by swapping variables between time series, leaving the variables the same individually, but combined into new time series (with other randomly selected variables). The code to create this type of surrogate data is as follows.

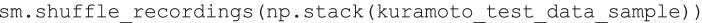



The sample must be a numpy array of shape (number_time_series, number_variables, number_time_steps). The function returns a surrogate sample with the same shape.

## Simulated and empirical demonstrations

Now that we have demonstrated how to compute the various metrics and take advantage of the functions of multiSyncPy, we next showcase the use of our package using the aforementioned two types of synthetic data as well as two existing empirical datasets. Doing so allows us to compare the methods for quantifying multivariate synchrony, and provide lessons learned from their application to different types of data. First, explorations of two types of synthetic data are presented. We used a stochastic autoregressive function, following the example of Gouhier and Guichard (2014), which produces variables that have temporal structure, but no above chance-level synchrony. Such variables can be stacked together to create a multivariate time series. Adding correlated noise to the variables in one of these synthetic time series gives a simplified illustration of the effects of unstructured noise on the synchrony metrics. Our other synthetic data comes from a stochastic Kuramoto model, which mathematically models a group of interacting oscillators, and in which the strength of coupling between oscillators can be systematically varied. With this data, it is possible to test our prediction that multivariate synchrony increases with the strength of the coupling parameter.

In addition to synthetic data, we use two datasets from real experiments. The ELEA corpus of recordings (Sanchez-Cortes et al., 2012) includes video recordings of small groups performing a team task, along with transcripts, questionnaires and annotations. We focus on the video recordings and use OpenPose (Cao et al., 2021) to extract information about the body posture of participants and how it changes over time. The synchronization of body movement is analyzed using various synchrony metrics as a case study. We also use data from another experiment investigating interactions in small groups (Gervais et al., 2013), which has previously been used to study the dyadic synchronization of body movement in triads (Dale et al., 2020), but not multivariate synchronization.

### Synthetic data results

**Fig. 2:**
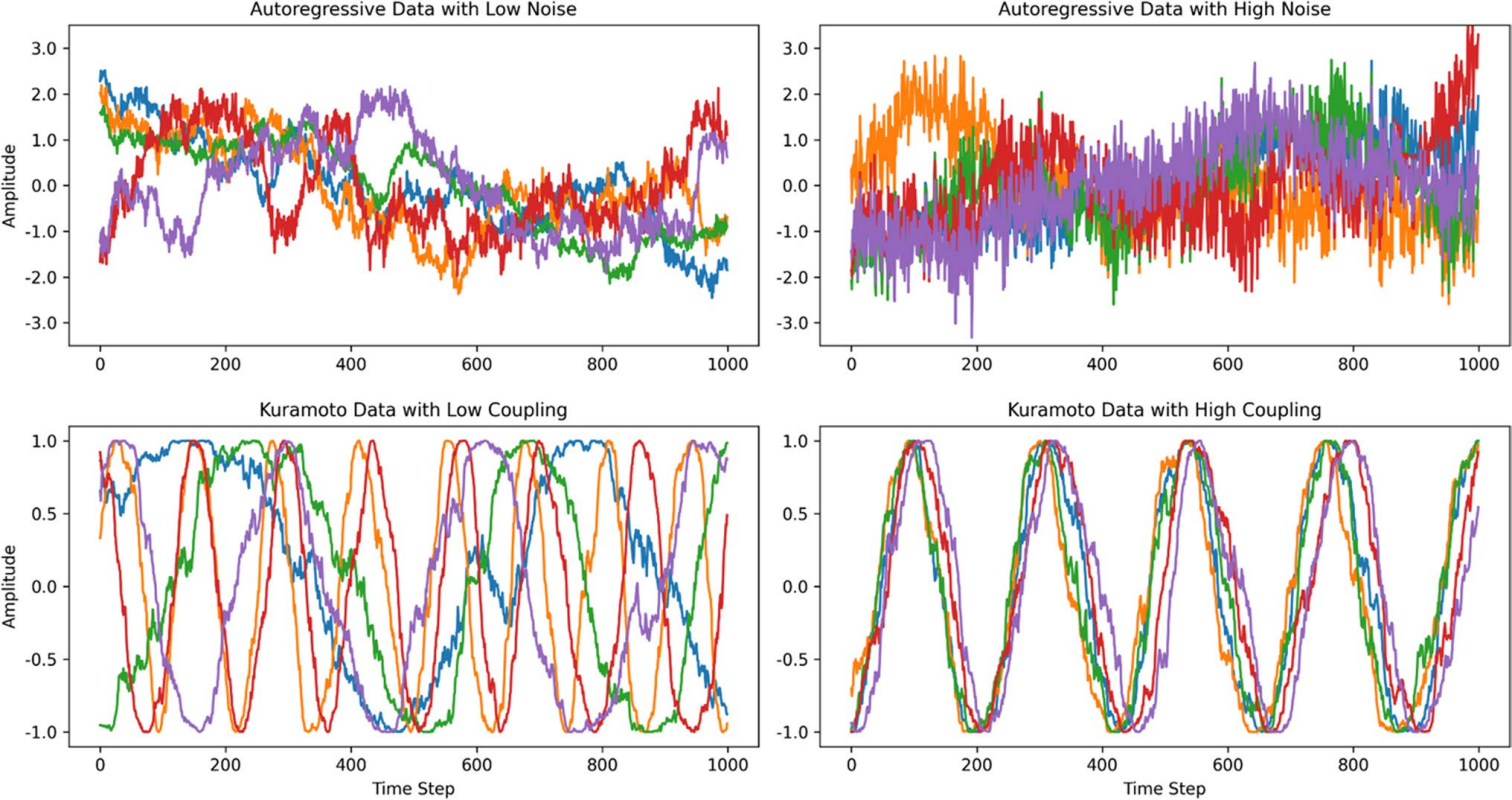
Synthetic data time series examples

As explained in the previous sections, the multiSyncPy package provides the ability to compute a range of metrics which assume varying forms of underlying coordination (e.g., matching of states, matching phase, etc.). However, so far it is not clear what the relative merits of the different methods are because most studies examining multivariate coordination employ only a single technique. Using synthetic data, we can compare the performance of these multivariate synchrony metrics by systematically varying simple parameters used to generate this data. Examples of our two types of synthetic time series (stochastic autoregressive process and Kuramoto oscillators) are visualized in Fig. 2.

#### Autoregressive data with correlated noise

The first type of synthetic data we examine comes from a stochastic autoregressive function. The data generated by this process has temporal structure, as each value in the time series is a product of the previous two (plus Gaussian noise), however the way that the values develop over time is unpredictable and a consequence of the Gaussian noise added at each time step. The process is described by the following equation, where *X*_*t*_ is the value of the process *X* at time *t*, *β*_0_, *β*_1_, *β*_2 _are fixed parameters and *ε*_*t*_ is Gaussian noise added at time *t*.

1 $${X}_t={\beta}_0+{\beta}_1{X}_{t-1}+{\beta}_2{X}_{t-2}+{\varepsilon}_t$$

The advantage of this is that two different time series generated in this manner should have temporal structure, but no above chance-level synchrony with one another. We generate five time series of length 1000 from the autoregressive function, and stack them alongside one another to act as the variables of a multivariate time series.

Following the example of Gouhier and Guichard (2014), an important addition to our method is that we also add a certain amount of correlated noise to the multivariate time series. Gouhier and Guichard contend that correlated noise, added to the signals in this way, might lead to the ‘false’ detection of synchrony occurring despite the fact that the underlying autoregressive processes are unrelated. According to this line of reasoning, how a metric is impacted by the presence of correlated noise is an important characteristic of how the metric performs.

We select a number from a Gaussian distribution at each time step, and then add this same number to each variable in the multivariate data. The relative contribution of the correlated noise to the final data is a parameter that we vary through the course of our experiments, i.e., we treat it as an independent variable. The variables in the synthetic data are each normalized to have mean 0 and variance 1, and the Gaussian distribution used to generate correlated noise has a mean of 0, but a variable standard deviation. To perform our investigations, we gradually increase the standard deviation of the correlated noise. It begins at zero, and increases in steps of 0.1 up to 1.0. At each value for the standard deviation of the correlated noise, we use the aforementioned procedure to create a sample of 500 time series, each containing five variables and 1000 time steps, and then compute the synchrony metrics. See Fig. 2 for example time series that might appear in a sample.

**Fig. 3:**
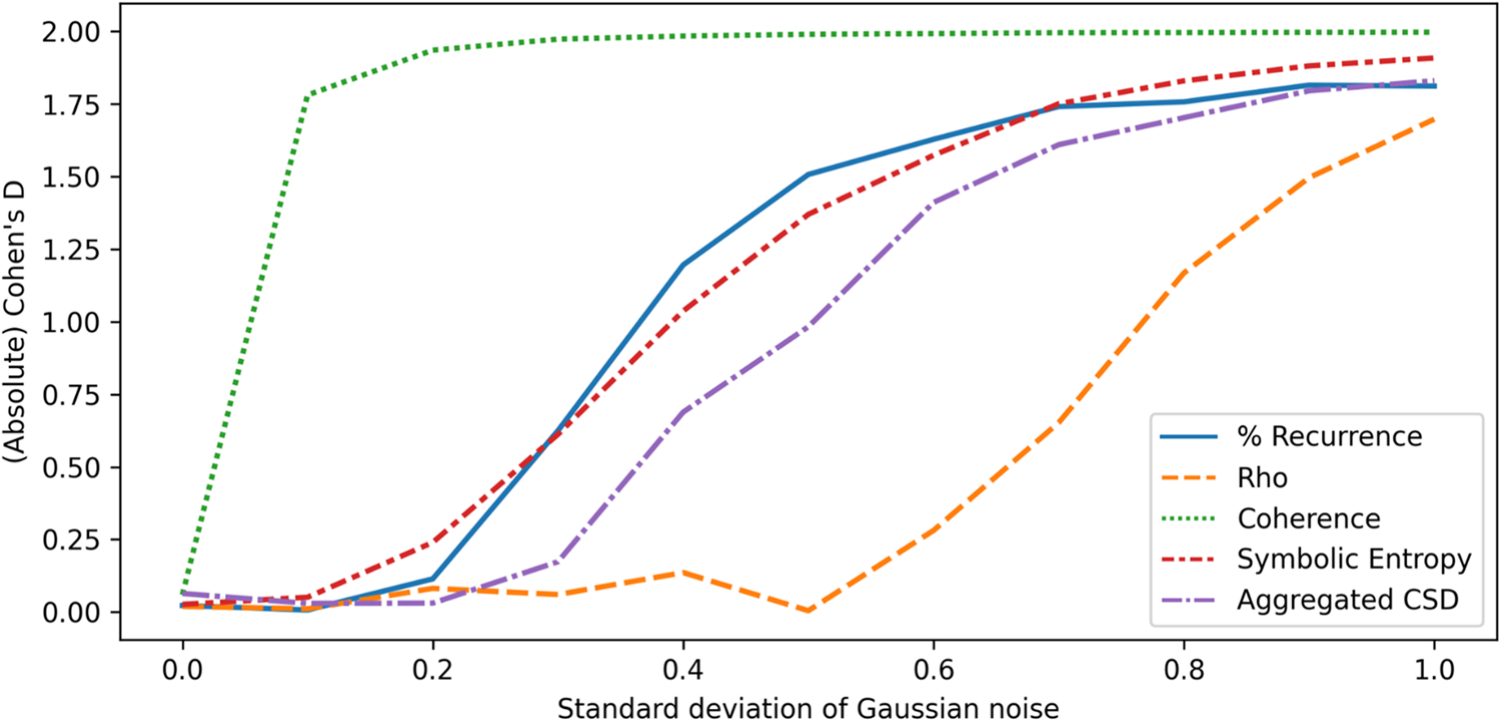
Absolute values for Cohen’s *d* when comparing synchrony scores on autoregressive data to surrogate data

**Fig. 4:**
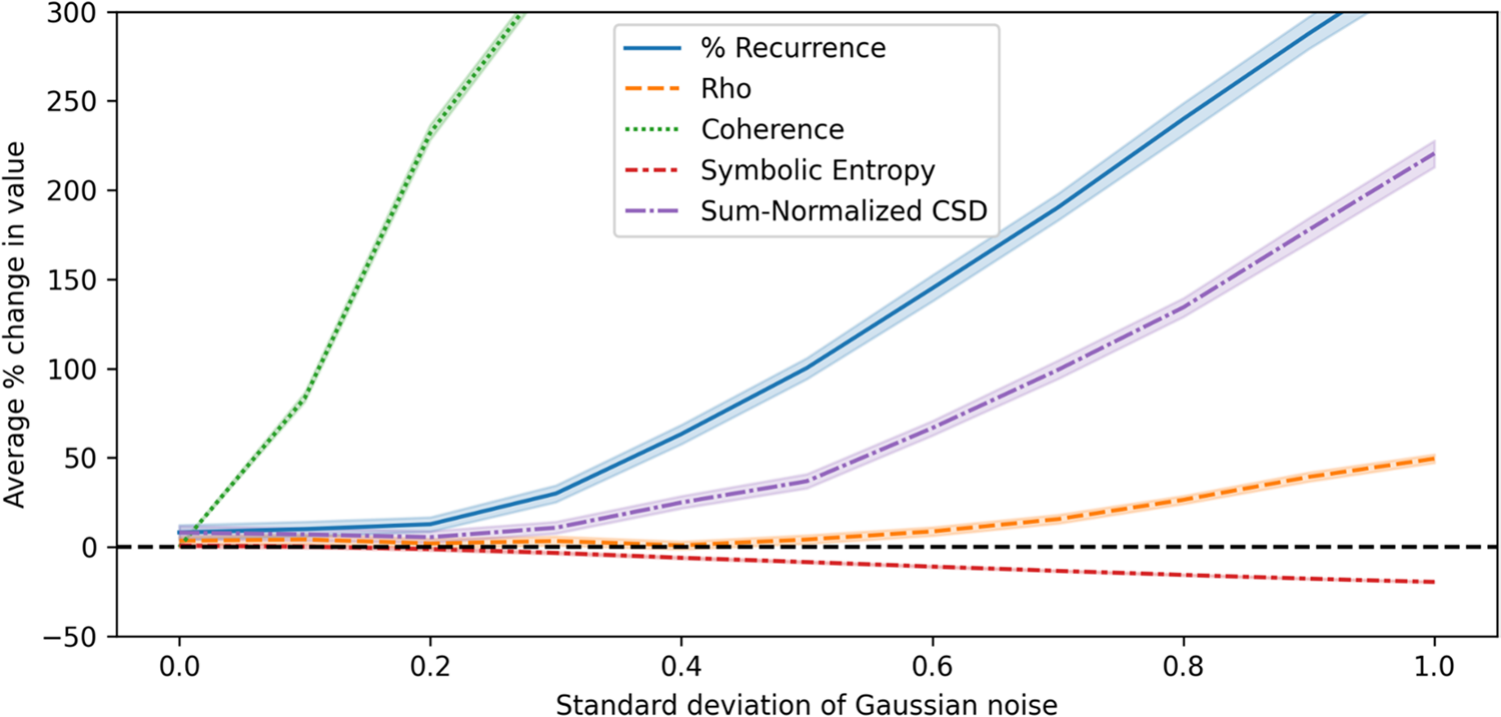
Change in synchrony scores on autoregressive data compared to surrogate with increasing levels of noise

To help us evaluate the observed values for each metric, we create surrogate data for each sample after adding correlated noise and re-calculate the metrics on these, providing ‘surrogate baseline’ results. The observed values without surrogation are compared to the surrogate baseline and we report Cohen's *d *and the percentage change to determine how robust the metrics are to changes in parameters of the synthetic data. We attempted both strategies mentioned in the previous section for constructing surrogate data, namely (1) shuffling windows of data, and (2) swapping variables between time series so that the variables have the same structure but appear in different time series. For the approach of shuffling windows, each variable was cut up into windows with length 1/10 of the total time steps and reordered. However, this produced unexpectedly large discrepancies between the main results and the surrogate results even when there was no correlated noise, while in this circumstance there is no synchrony expected between variables in the original time series or the surrogate baseline (since the variables come from unconnected autoregressive processes), and so the outputs should not differ noticeably. For this reason, we report the results using the second type of surrogate data, in which variables are swapped. For this type of surrogate data, the results were as expected when no correlated noise had been added. The results are presented in Figs. 3 and 4, which respectively show the (absolute value of) Cohen’s *d* and the average percentage increase in each metric, compared to the surrogate baseline where variables had been swapped between time series after adding correlated noise.

Using the outputs of this investigation, we are able to compare how synchrony metrics perform as the standard deviation of the correlated noise is increased, and highlight some important differences. All of the metrics are evidently affected once the standard deviation of the correlated noise becomes sufficiently high. As shown in Fig. 4, the coherence metric appears to be affected soonest, increasing quickly over the corresponding baseline values. Coherence and percentage of recurrence exhibit dramatic rises as a response to the correlated noise, ending up with such high values compared to the baselines that it is impractical to plot fully in the figure. Coherence reaches a plateau of approximately 500% average change in value when scores reach the maximum value of 1.0 while baseline values remain essentially unchanged. Our proposed sum-normalized CSD metric takes longer to rise over the baseline, compared to the closely related coherence metric, although eventually this also becomes strongly affected by the correlated noise. The reason for this difference is that the noise tends to affect all the frequency components of the signals, even when it is added in a small degree. The coherence score is much more influenced by the number of frequency components at which there is high cross-spectral density, whilst the sum-normalized CSD is also sensitive to the amplitude of the frequency components and therefore is less affected when there is only a small degree of synchronization (due to correlated noise) in many of the frequency components.

Relative to the other metrics, rho appears to stay close to zero for the longest, and has one of the lowest increases over the baselines when the correlated noise has its highest standard deviation. Unlike with coherence, the rho metric did not reach values close to its maximum of 1.0. One reason rho is less likely to be impacted by correlated noise is that this metric is based upon phase, which quantifies progression through some structured pattern; since the added noise does not have temporal structure, it may have a limited impact on the extracted phase, and therefore affect rho less than it affects the other metrics.

When interpreting the percentage change results, it is important to note that, while the surrogate baseline values for coherence and rho remained largely consistent while changing the correlated noise and then shuffling variables to create surrogate data, the opposite was true for recurrence. The recurrence of the first baseline was on average 13.6% without correlated noise, but fell to 1.1% when the standard deviation of the noise was 1.0. This may help to explain the larger over-the-baseline increases observed in the recurrence. Symbolic entropy appears to have remained close to zero for longer than recurrence and coherence while increasing the standard deviation of the noise, but not for as long as rho. At no point was the change over the baselines particularly large for symbolic entropy. When interpreting the symbolic entropy, it is worth noting that there is a theoretical minimum of roughly 1.1 and a theoretical maximum value of 5.5 when using five variables. This provides some limitation on how much the observed entropy can increase over a baseline, which is not the case for the other metrics.

Before moving on, a note of caution is that these results give a limited impression of how the metrics will perform in real scenarios. The autoregressive data, especially when we use a high parameter value for noise, adds correlated random numbers at each time step, which may be quite different from what would be expected with real sources of noise. Real sources might occur infrequently rather than having a consistent impact over all time points or have a more distinctive spectrum. Nevertheless, these investigations with unstructured but correlated noise can help to give an initial characterization of the different metrics in multiSyncPy.

#### Kuramoto model data

Next, we quantify the impact of increasing the coupling strength of oscillators in a Kuramoto model on the synchrony metrics. We assume that data generated from models where the coupling is higher will have higher levels of synchrony on average, and the following results show how well our metrics reflect this assumed trend. For each variable *i* in the model (which we base on the equations of Acebrón et al., 2005), the update rule for the phase of the variable is given below. *θ*_*i *_is the phase/angle of the variable and *ω*_*i *_is the natural frequency of the variable. K is the coupling strength parameter, which is shared across the system. *ψ *is the average phase. If each variable is represented as a point along the circumference of the unit circle, then *r* is the distance of the centroid from the origin. The final term, *ξ*_*i*_(*t*) denotes the random noise added at time *t*.

2 $${\overset{\acute{\mkern6mu}}{\theta}}_i={\omega}_i+ Krsin\left(\psi -{\theta}_i\right)+{\xi}_{i(t)}$$

**Fig. 5:**
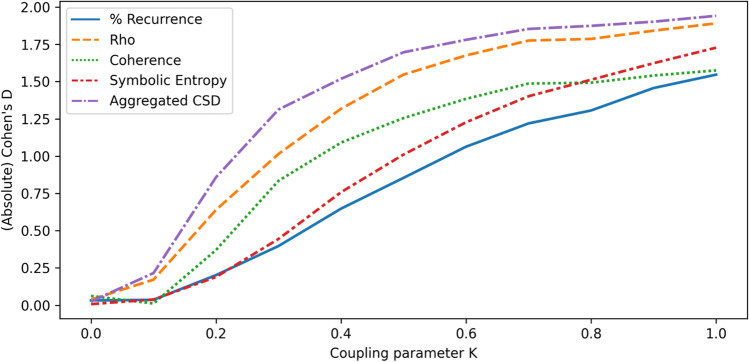
Absolute values for Cohen’s *d* when comparing synchrony scores on Kuramoto data to surrogate data

**Fig. 6:**
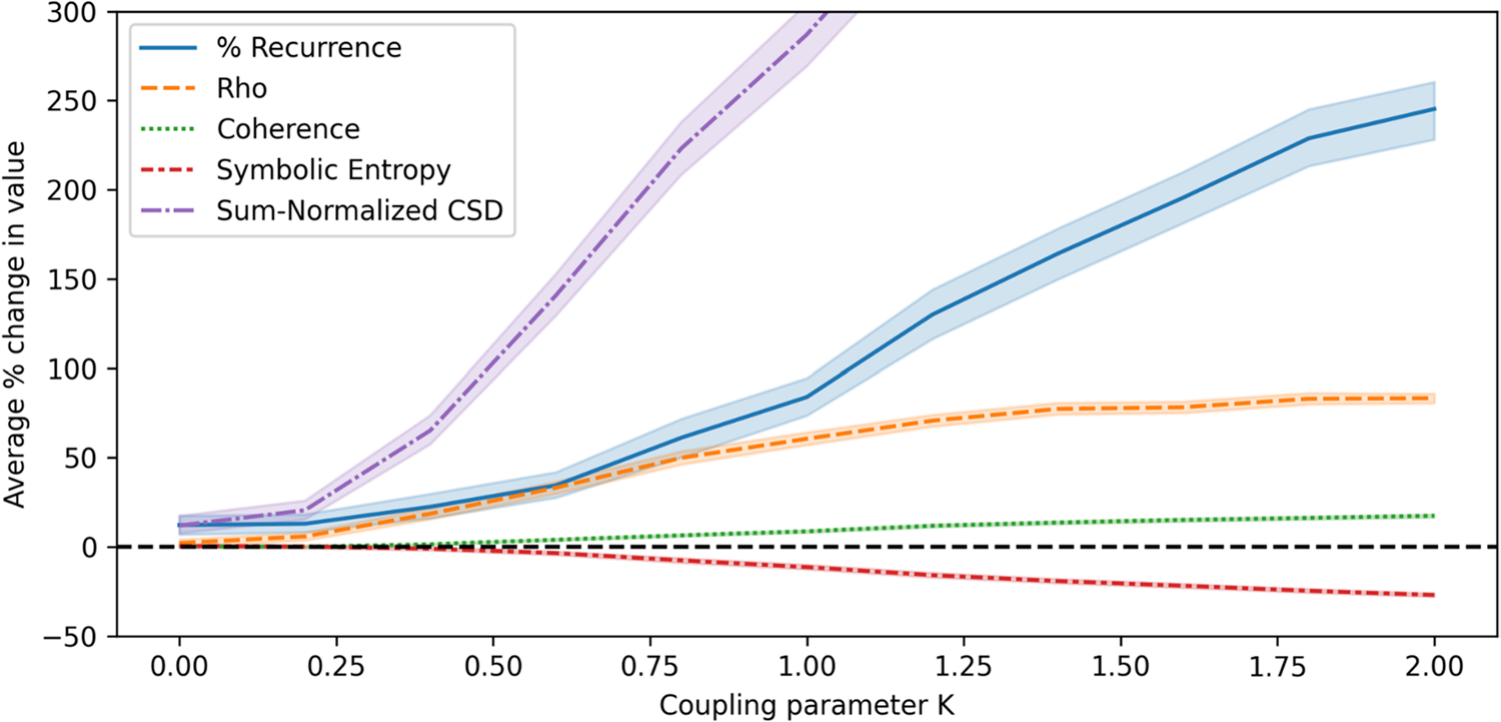
Change in synchrony scores on Kuramoto data compared to surrogate at increasing levels of coupling strength

We systematically increase the coupling strength parameter K, from 0.0 to 2.0 in steps of 0.2 and investigate the impact on the synchrony metrics. For each setting of K, we create a sample of 500 Kuramoto models and generate a multivariate time series with five variables and 1000 time steps. For each model in the sample, we choose a different set of natural frequencies for the oscillators sampled from an exponential distribution. This is important for the sake of our surrogate baseline where variables are swapped across time series, remaining the same but ending up in different time series, since high levels of synchrony could be observed in the surrogate data simply because the same natural frequencies are repeated across variables in the sample. Figures 5 and 6 show how the synchrony scores vary as the coupling strength is increased, compared to a surrogate baseline made by swapping the very same variables across time series in the sample.

Using these results to compare the metrics, we find that all metrics increase as the coupling in the Kuramoto models is strengthened. Figure 5 shows that the estimated effect increases similarly for all metrics, although Fig. 6 shows that the magnitude of the change over the baseline can differ quite considerably.

In Fig. 6, the potential effect of Gaussian noise on the coherence metric is exposed, with the coherence scores barely rising above the baseline, even with strong coupling between oscillators. This is due to the fact that coherence at frequencies where the amplitude is high are treated equally with coherence at low-amplitude frequencies, when averaging across frequency components. In our Kuramoto data, each signal is a combination of a sinusoidal progression and a small amount of added Gaussian noise. Synchronization of the main sinusoidal component occurs at a single frequency, while the unsynchronized Gaussian noise (being white noise) affects the signal at all frequencies. Due to the normalization that occurs when calculating coherence at each frequency component (which removes information about amplitude), the presence of white noise at many frequencies ends up being dominant in the final averaged coherence score. The sum-normalized CSD metric we propose is better able to handle this issue, by adjusting the way that normalization is applied to the cross-spectral density. This is demonstrated by the fact that it rises quickly over the baseline as the coupling strength between oscillators is increased. Note that despite this issue with coherence, Fig. 5 indicates that an effect is observed when comparing the coherence scores to the baseline, meaning that the metric may still be useful in analyzing experimental data.

Rho seemingly exhibits an increase over the baseline more quickly than some of the other metrics when coupling strength becomes larger, suggesting it does detect synchrony well in this context. The percentage of recurrence also seems to be responsive to coupling strength when looking at the percentage increase over the baseline, however the estimated effect size increases slightly more slowly than with the other metrics. Symbolic entropy showed lower increases over the surrogate baseline for smaller coupling strengths, but still demonstrated increases when the coupling strength was higher. Overall, the metrics fit the assumption that increased synchrony should be detected when the coupling between oscillators is increased.

Using this type of synthetic data from Kuramoto models, it is possible to investigate various types of validity for our metrics. First, we can gain an insight into convergent validity by calculating the correlation between the metrics, since they are all expected to measure some form of multivariate synchrony. We do this across all Kuramoto time series generated according to the above procedure. With the same data, we also consider whether the multivariate metrics have concurrent validity with a metric that reflects the related concept of dyadic synchrony. We chose cross-correlation as a standard dyadic metric (Schoenherr et al., 2019) with a lag of 0, and averaged across all pairs within a time series to obtain a single value per time series. Pearson correlation coefficients are presented in Table 1.

**Table 1:** Pearson correlation coefficient between multivariate synchronization metrics

	Rho	Coherence	Symbolic entropy	Sum-normalized CSD	Mean dyadic correlation
% Recurrence	.74	.82	– .91	.67	.88
Rho		.69	– .90	.92	.88
Coherence			– .76	.68	.80
Symbolic entropy				– .83	– .93
Sum-normalized CSD					.85

The absolute values of the correlation coefficient are all above .66, which Schoenherr et al. (2019) classified as ‘high’ correlation when comparing dyadic synchrony metrics, suggesting that there is substantial convergent validity between the different measures. Coherence has slightly lower scores than the others, perhaps due to the effect that Gaussian noise has on the metric when using this type of synthetic data (as described earlier). Nevertheless, these correlations are generally high and give support for convergent validity amongst the metrics.

Additionally, it is possible to examine how well each multivariate metric is suited to predicting the known coupling parameter of the Kuramoto models, providing an insight into criterion validity. Figure 6 shows that across all metrics, there is a nearly perfect correlation (|*r*(9)| > .95) between the coupling parameter and the average synchrony score of the time series generated using that coupling parameter. This provides strong evidence of criterion validity, in that all metrics increase when synchrony in the form of coupling is increased.

It is worth noting that this synthetic data may not resemble all of the important aspects of empirical signals as the Kuramoto data we generated assumes that the signals all come from periodic oscillators with equal and constant coupling.

### Empirical data results

To complement the investigation of synthetic data, which allows us to vary key parameters of the data and examine the consequences, we also examine data from empirical studies, which have more complex and realistic properties, but at the cost that we do not know what parameters are driving it. We present two case studies using openly available data from the ELEA corpus (Sanchez-Cortes et al., 2012) and a study of triadic interaction (Gervais et al., 2013; Dale et al., 2020).

Using these real-world data sets, we are able to showcase how multiSyncPy is used to perform novel scientific work, while also presenting new results from an analysis of bodily synchrony in two different tasks. In fact, our results on the ELEA corpus are the first to our knowledge to investigate bodily synchrony in this data. Through the case studies, we are able to provide ‘lessons learned’ from initial usage of the package. This makes it possible to describe the difficulties that real-world data, as opposed to synthetic data, can present. It also serves to highlight that it can be difficult to detect group-level synchrony, including in a case study where dyadic synchrony had been observed previously.

#### ELEA corpus

The ELEA corpus (Sanchez-Cortes et al., 2012) contains video recordings and transcripts from groups of three or four people performing a disaster survival task where the group must rank the importance of a list of items given a disaster scenario. The corpus also includes details of the rankings that individuals came up with individually and the group rankings they agreed upon, plus personality questionnaires, and ratings of dominance and leadership provided by experts.

We use the videos included in the corpus to investigate postural synchrony within the teams. First, key points of the participants’ bodies were extracted for each frame of video using OpenPose (Cao et al., 2021). Then, we select a subset of key points to represent posture. Specifically, we use key points 0, 1, 2, 5, 15, 16, 17 and 18 from model ‘BODY_25’, which correspond to the nose, neck, right and left shoulders, right and left eyes, and right and left ears. These were chosen on the basis that they reflect postural information while being visible in the ELEA video recordings, which show participants from above the waist. Calculating the Euclidean distance for each key point across frames and then averaging across key points gives a single postural movement signal for each participant; the three or four participants together make a multivariate time series. This method is commonly used for dimensionality reduction of motion capture data and is sometimes referred to as an interpoint distance time series (Davis et al., 2016) or displacement time series (Borjon et al., 2018).

**Fig. 7:**
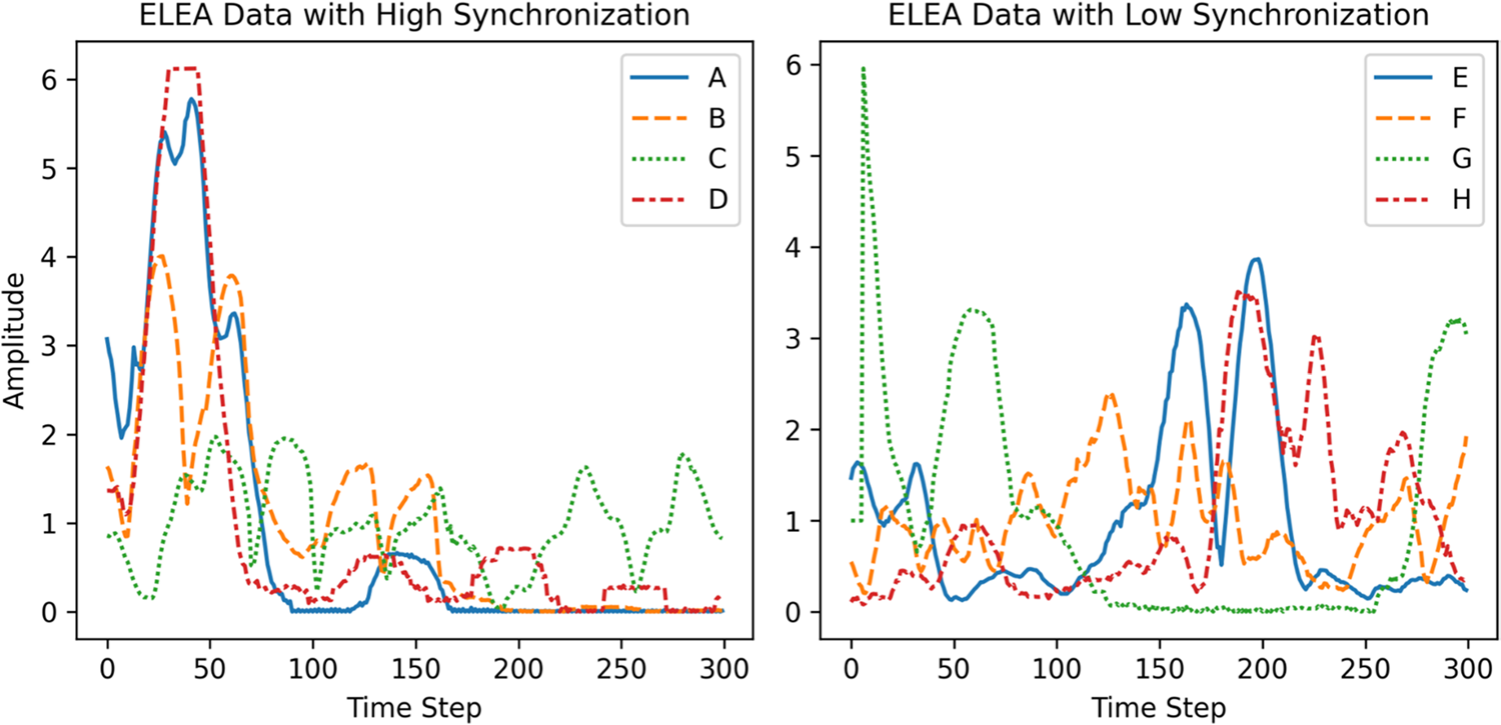
Example movement data extracted from ELEA from two meetings each with four participants, with participants indicated by line style and color

Additionally, there are occasional jumps in the positions of key points estimated with OpenPose, for example where the participant moved out of view of the camera, or another person appears briefly, which leads to sudden large changes in the Euclidean distances. These points were identified using outlier detection (if they possessed a z-score greater than 5) and replaced using linear interpolation. This affected only 0.3% of data. Because the recordings are different lengths, we take 14,000 frames from the middle of each recording, to produce a dataset with greater consistency and with which it is easier to swap variables across recordings to produce a surrogate baseline. Because we are interested in the videos, we use the ELEA-AV sub-corpus, which includes all the sessions which were video-recorded, amounting to 26 different teams. From these, the 20 recordings with four members in a team were selected for use, so that each multivariate time series would contain the same number of variables. Windows containing 300 time steps of data (equating to 10 s of recording) with high and low levels of synchronization are displayed below in Fig. 7 (each letter/line refers to postural movements from different group members).

Our first goal in investigating the data is to identify whether synchrony occurs at above the level expected by chance. Our first option in this regard is to apply the statistical test of Frank and Richardson on the Kuramoto order parameter. This method compares the observed data to a null hypothesis stating how a sample of values is expected to be distributed. This works for the Kuramoto order parameter. For the other metrics included in the package, the expected distribution of values in a sample is not known, and so we use surrogate data to gain an understanding of whether observed synchrony is above chance level.

In general, the test of the Kuramoto order parameter offers one way to examine levels of synchrony within a sample of recordings. However, this is predicated on the data being suitable for such analyses. The test is based upon the Kuramoto model of oscillating components, in which the components follow sinusoidal progressions and have a uniform distribution of phase values. As can be seen from Fig. 7, the ELEA data is characterized by short spikes or peaks in movement occurring with a background of no motion otherwise. Using this data, the phase values extracted by the Hilbert transform have a skewed rather than uniform distribution (due to the periods of stillness between spikes which have no progression).

To demonstrate the point, we examine what happens if one does perform the test of the Kuramoto order parameter on the movement data. For the 20 recordings of four variables, *t*(19) = 51.7, *p* < .001, 95% CI [.49, .61], Cohen’s *d* = 10.4, demonstrating a strong effect. However, we also find that when shuffling variables between time series to create a surrogate baseline in which synchronization is not expected, we achieve very similar results: *t*(19) = 46.5, *p* < .001, CI = [.45, .56], Cohen’s *d* = 10.4. This acts as a word of caution and a ‘lesson learned’ from our empirical case study, confirming that the test of the Kuramoto order parameter must be used on data from which appropriate phase information can be extracted, otherwise the results may be highly inflated. The distribution of phase values can be inspected to see if it is (roughly) uniform before performing the test. These findings also suggest that there is value in having access to multiple methods for analyzing synchrony, which is what multiSyncPy offers, since one method may overcome the limitations of another method and provide a way to identify inconsistent results. Further investigations to determine how both the type of data and the extracted phase information impact the test of the Kuramoto order parameter, using synthetic data and alternative methods for extracting phase, is presented in our Appendix I.

Our next focus is the performance of the remaining synchrony metrics compared to a surrogate baseline. We take the same data and compute the percentage of recurrence (using a radius of 0.4 to determine recurrent points), cluster-phase rho, coherence, sum-normalized CSD and symbolic entropy. Then, the variables are shuffled across recordings to produce a surrogate baseline and the metrics are re-computed for comparison. The mean values of the different metrics are presented in Table 2, along with standard deviations, the results of a Welch’s independent samples *t* test, and estimated effect sizes. All of the metrics show some differences that are consistent with the hypothesis that synchrony is greater in the real compared to the surrogate data, and *t* tests suggest significance across all metrics except recurrence (as shown in Table 2) after applying a Bonferroni adjustment for the fact that we are running five tests, which lowers the significance level to .01. For all metrics, the differences appear quite small in terms of absolute difference, although the effect sizes for rho, coherence, symbolic entropy and sum-normalized CSD are typically considered large. These results give some indication that global multivariate synchrony may have occurred during the team task.

**Table 2:** Synchrony metrics on ELEA recordings

Metric	M	SD	M (surrogate)	SD (surrogate)	*df*	*t*	*p*	Cohen’s *d*
Recurrence (%)	5.00	2.42	3.59	0.81	23.3	2.48	.021	0.74
Rho	0.57	0.03	0.52	0.01	26.4	7.46	< .001	1.52
Coherence	0.03	0.01	0.02	0.00	19.8	3.92	< .001	1.06
Symbolic entropy	4.30	0.10	4.38	0.01	19.2	– 3.31	.004	– 0.93
Sum-normalized CSD	0.03	0.02	0.01	0.00	19.4	3.72	.001	1.02

**Fig. 8:**
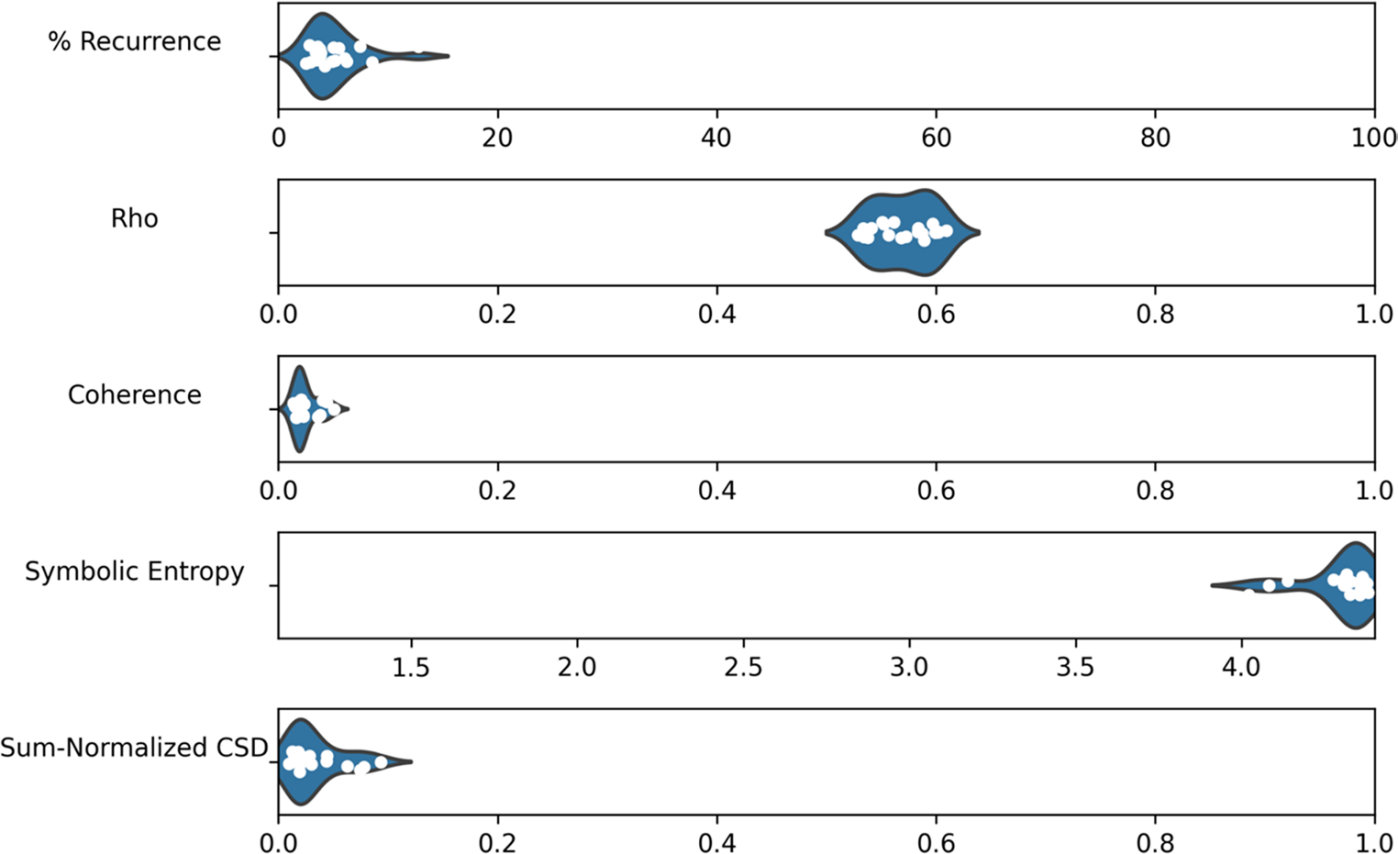
Distribution of scores for each metric, with scales determined by theoretical minimum and maximum

The relative distribution of the synchrony metrics across ELEA meetings is shown in Fig. 8, giving an indication of variability in the data. Because the range and units of each measure varies, each has its own scale determined by the theoretical minimum and maximum. Recurrence values are generally close to 5%, which is to be expected since the radius used to decide recurrence was chosen specifically to produce around 5% recurrence. Coherence and sum-normalized CSD have low values relative to the theoretical maximum, although this is highly influenced by the fact that the time series have 14,000 time steps, allowing for the extraction of many frequency components, while it would not be expected that synchronization occurs at all of these frequencies. Symbolic entropy scores tend to be close to the theoretical maximum, but still demonstrate variability.

We also analyzed the data dividing each meeting into smaller, non-overlapping windows, following the example of (Dale et al., 2020). We used 300 frames for a window, corresponding to 10 s. Since OpenPose is unable to accurately extract key point locations in every frame, which may impact the reliability of the results, we select windows where OpenPose reports a confidence of 100% when extracting key points in all frames. The number of windows obtained from a meeting can vary, according to the length of the meeting and the proportion of frames where OpenPose reports less-than-perfect confidence. The number of windows from a meeting varies between 9 and 79, with a median of 48.5.

We then identify the maximum values of synchrony across all windows for a meeting, and use this score to summarize the entire meeting (Dale et al., 2020). This is motivated by the notion that synchrony may not occur consistently across a group meeting and instead has time-varying properties (Likens & Wiltshire, 2020; Mayo & Gordon, 2020; Wiltshire, Steffensen, et al., 2020b). With this method it should be possible to detect the presence of synchrony in general by looking at the maximum across time segments of a meeting (or observed period of interaction). Like with our previous analyses, we also create a surrogate baseline by swapping variables across windows, and then extracting maxima per meeting. The number of meetings and the number of windows per meeting is kept the same in the surrogate baseline.

There are a few additional things to note about how the different metrics are calculated on this data. First, extracting the maximum recurrence across windows in a meeting led to particularly high values, being above 30% in some cases. This implies that the radius used previously to decide when points are recurrent might be too large, and so we reduce the radius for this piece of analysis from 0.4 to 0.3 (see (Wallot & Leonardi, 2018) for guidance on setting the radius parameter for recurrence quantification analyses). Second, the highest point of synchronization is reflected by the lowest entropy, and so the minimum value rather than the maximum is used for that metric. Because the sequences consist of only 300 time steps, we reduced the window length to 75 (from the scipy default of 256) when using Welch’s method to determine power spectral density within the coherence calculation. This allows for multiple overlapping windows to be used in the calculation, which improves the estimation of spectral density and leads to usable results that are reported here. The results for the actual observations and the surrogate baseline are shown in Table 3. The first two columns show the mean value across meetings, with the standard deviation in parentheses. The next columns show the results from Welch’s independent samples *t* tests, and the estimated effect size.

**Table 3:** Synchrony metrics based on maximum value from windows per ELEA recording

Metric	M	SD	M (surrogate)	SD (surrogate)	*df*	*t*	*p*	Cohen’s *d*
Recurrence (%)	6.70	3.10	6.43	2.96	37.9	0.28	.780	0.09
Rho	0.83	0.05	0.82	0.05	37.8	0.87	.391	0.28
Coherence	0.28	0.04	0.26	0.03	35.6	1.02	.316	0.32
Symbolic entropy	3.00	0.08	3.00	0.12	34.4	– 0.33	.744	– 0.11
Sum-normalized CSD	0.31	0.07	0.28	0.05	34.6	1.83	.076	0.56

**Fig. 9:**
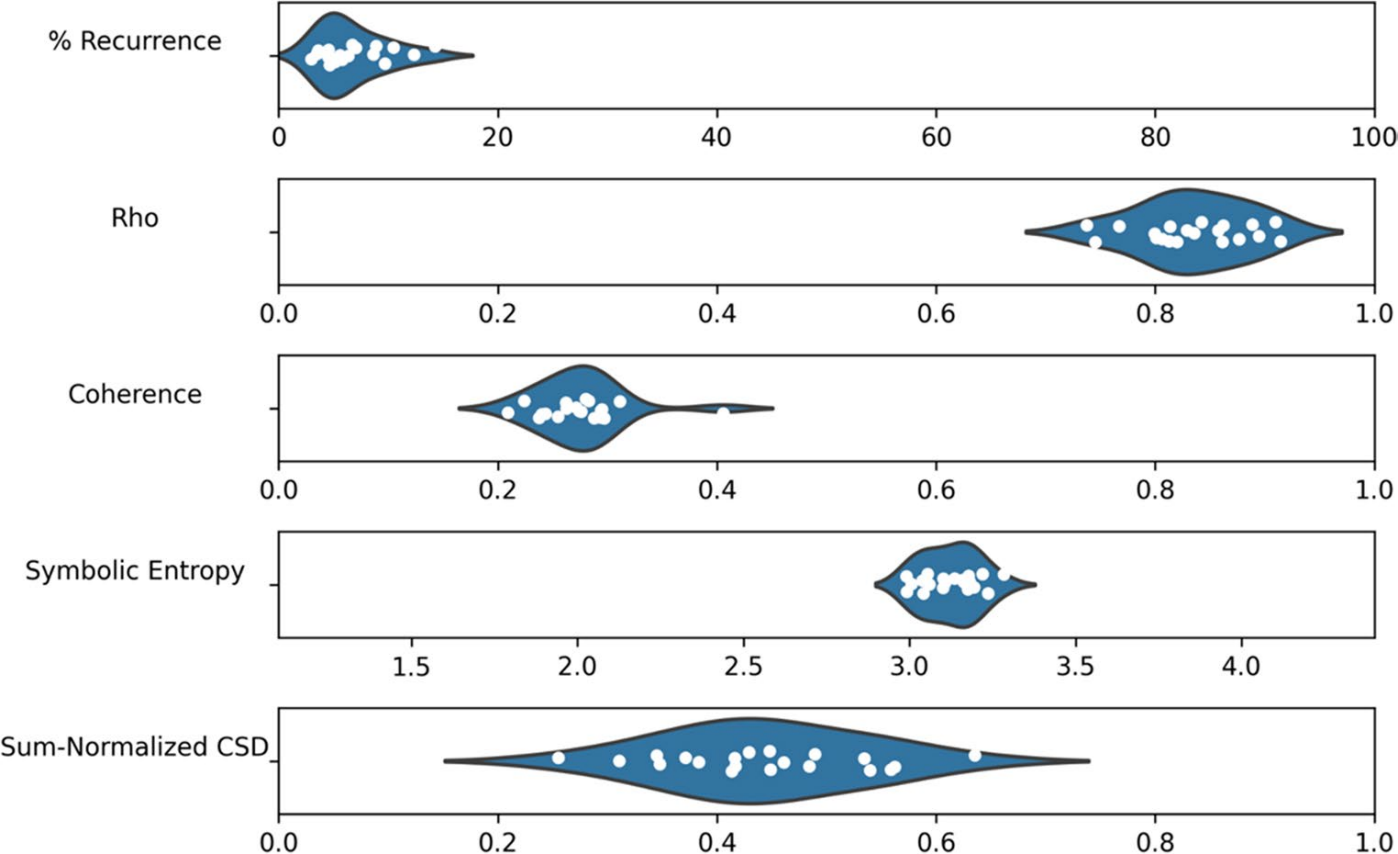
Distribution of scores for highest synchrony across windows per ELEA meeting, with scale determined by theoretical minimum and maximum

Consistent with our previous results, we find that the synchrony values show an increase over the surrogate baseline, except for entropy which changes negligibly, giving us some added confidence that synchrony is still present after excluding frames where OpenPose had low confidence. However, using independent-samples *t* tests with a Bonferroni adjustment to reduce the significance level to .01 does not suggest that there is statistical significance to any of these differences. All of the metrics showed increased synchrony scores compared to the baseline, but the increases are small and not statistically significant. Figure 9 shows the relative distribution of the synchrony metrics for this data, i.e., using the value reflecting highest synchronization from the windows for each ELEA meeting. Compared to the 14,000 time step data used previously, the variability observed is greater with these windows of 300 time steps.

Previously, we examined the convergent validity between different multivariate synchrony metrics and an averaged dyadic measure when using synthetic Kuramoto data. With the ELEA data, we are able to examine convergent validity when using empirical data. The correlations between metrics across all 1222 windows of 300 time steps are presented in Table 4.

**Table 4:** Pearson correlation coefficient between metrics, windows of ELEA movement data

	Rho	Coherence	Symbolic entropy	Sum-normalized CSD	Mean dyadic correlation
Recurrence (%)	.48	.38	– .40	.23	.37
Rho		.33	– .27	.26	.58
Coherence			– .02	.41	.47
Symbolic entropy				-.04	-.01
Sum-normalized CSD					.41

The correlations presented here are generally of lower magnitude than when using Kuramoto data, however they also show a similar positive correlation between different metrics, with the exception of symbolic entropy which decreases when synchronization is higher, as is expected. Where the magnitude is greater than .05, the results are significant. This gives some additional confirmation of convergent validity even when using empirical data.

#### Triadic synchrony dataset

**Fig. 10:**
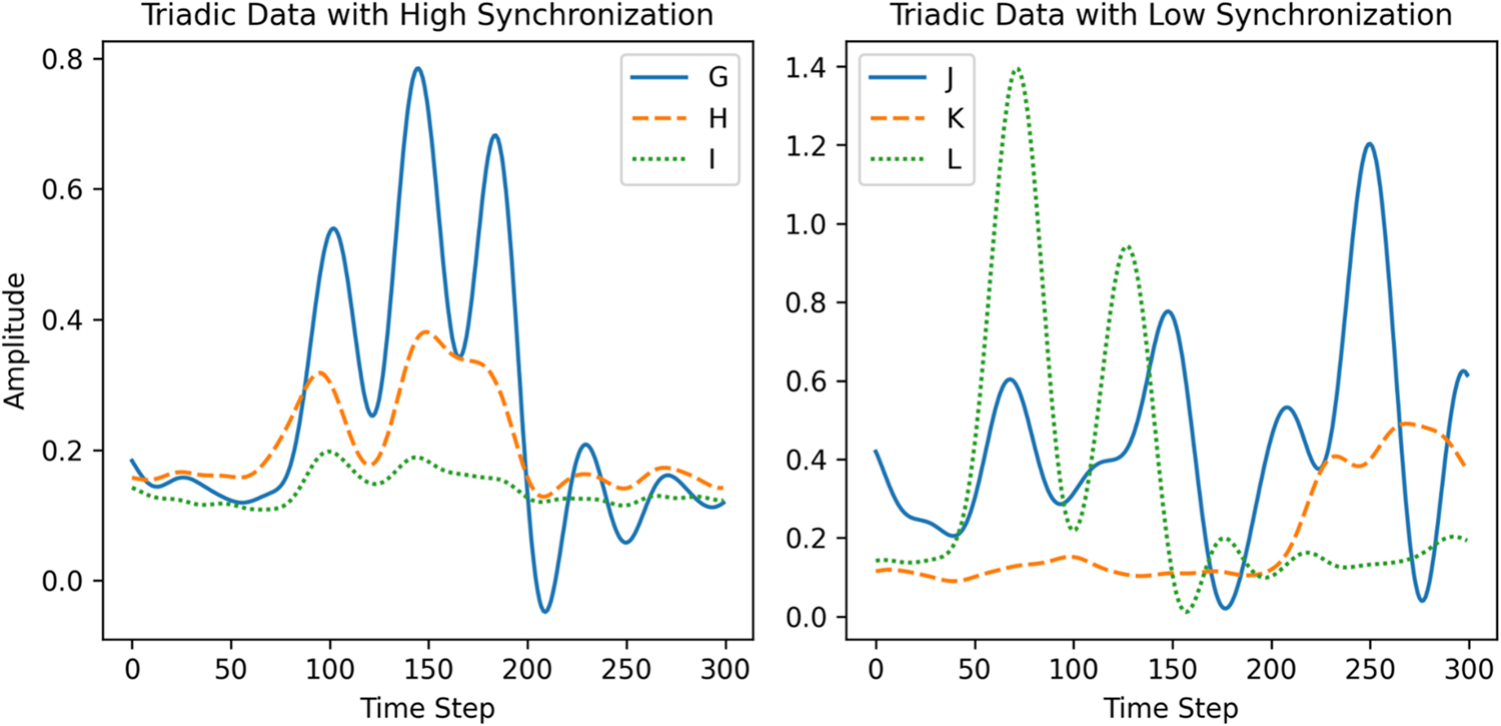
Example triadic movement data with high and low synchronization

A final component of our investigations into the performance of our metrics was to apply our analysis method to a dataset that has previously been used to study synchrony. We use the data analyzed by Dale and colleagues (Dale et al., 2020; Gervais et al., 2013) in their investigation of cross-correlation in triads. Their work looked at the interactions between groups of three participants in a period of open-ended conversation, with no goal or topic provided as part of the experiment. From video recordings, they extracted information about body movement and examined it for evidence of synchronization. Instead of using OpenPose, an ‘optical flow’ method was applied that measures the average change in pixel intensity over time to provide a proxy for body movement (Barbosa, 2017; Latif et al., 2014). The analysis focused on cross-correlation amongst the dyads that comprise a triad, and whether these cross-correlations themselves synchronized. Our approach is different in that it directly investigates synchrony as a group-level construct. Windows containing 300 time steps of data (equating to 10 s of recording) with high and low levels of synchronization are displayed below in Fig. 10 (with the movements of each participant corresponding to a different line/letter).

We apply the same pre-processing as used in the original work, specifically a low-pass filter at 0.05 of the Nyquist frequency followed by excluding the first 200 frames from each meeting. The meetings are then split into non-overlapping windows of 300 frames, representing 10 s duration. We select the maximum observed synchrony across the windows for each meeting, and compare these per-meeting maxima to those observed in a surrogate baseline, where the variables have been swapped across recordings. In our surrogate baseline, we use a number of meetings and windows equal to those used in the main results. Consistent with our analysis of windows of ELEA data, a radius of 0.3 was used for mdRQA, the minimum rather than the maximum entropy per meeting is used, and the window size when computing power spectral density as part of the coherence calculation was reduced to 75. The mean values of the various synchrony metrics are presented in Table 5, along with standard deviations and results of Welch’s independent samples *t* tests and estimates of effect size.

**Table 5:** Synchrony metrics on triadic meeting data

Metric	M	SD	M (surro- gate)	SD (surro- gate)	*df*	*t*	*p*	Cohen’s *d*
Recurrence (%)	12.82	4.52	11.74	3.43	63.4	1.13	.264	0.27
Rho	0.91	0.03	0.89	0.04	67.2	1.70	.095	0.40
Coherence	0.59	0.08	0.51	0.08	67.9	3.62	< .001	0.80
Symbolic entropy	2.50	0.10	2.55	0.11	64.3	– 2.45	.017	– 0.57
Sum-normalized CSD	0.50	0.07	0.46	0.08	66.9	3.26	.002	0.73

The percentage of recurrence is higher in the real data compared to the surrogate baseline, consistent with the hypothesis that synchronization occurs during the conversations. The change is still quite small however, so this provides only weak evidence of multivariate synchrony. Similar to recurrence, symbolic entropy is slightly lower on the real data as would be expected if a small amount of synchrony is present in the data. Applying a Bonferroni adjustment to lower the significance level to .01 for our independent-samples *t* tests, recurrence, symbolic entropy and the cluster-phase ‘rho’ metric do not exhibit a significant change over the baseline. Coherence and the related sum-normalized CSD do suggest the presence of synchrony, with the difference from the surrogate baseline being significant according to a Welch’s independent samples *t* test. The results are mixed, with only two related metrics suggesting a significant change from the baseline, but there is some limited evidence of the presence of group-level synchronization.

**Fig. 11:**
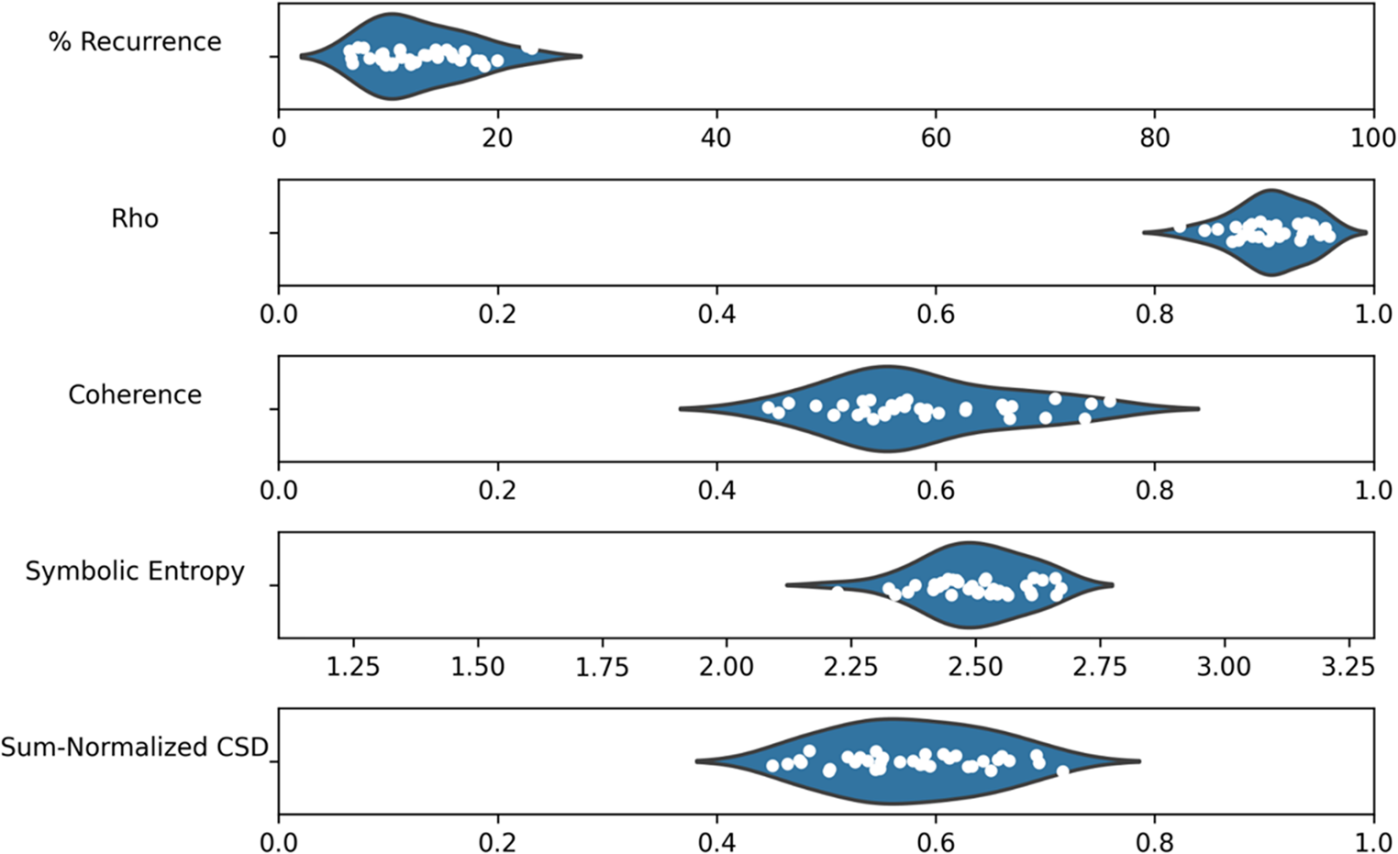
Distribution of scores for highest synchrony across windows per triadic meeting, with scale determined by theoretical minimum and maximum

For most of our metrics, the average increase over the baseline is smaller for our metrics than was found in the investigation of cross-correlations in the original paper (Dale et al., 2020). This may be because the earlier work used a pairwise concept of synchrony rather than a full triadic/group-level metric. It may simply be less common for all three members of a group to synchronize together than it is for two individuals to synchronize, hence the difference in our results compared to the average cross-correlations reported in the original work (Dale et al., 2020). The relative distribution of the synchrony metrics is shown in Fig. 11, giving an indication of variability when using the triadic data.

As with the data sets described previously, it is possible to examine the correlations between metrics on this empirical data. Table 6 displays Pearson correlation coefficients when using the full 1960 windows available from the triadic meetings. The metrics have small to medium correlations, all of which are significant at *p* < .001, giving further evidence of convergent validity between the metrics included in our package.

**Table 6:** Pearson correlation coefficient between metrics, windows of triadic movement data

	Rho	Coherence	Symbolic entropy	Sum-normalized CSD	Mean dyadic correlation
Recurrence (%)	.40	.17	– .33	.15	.22
Rho		.33	– .44	.32	.59
Coherence			– .20	.41	.47
Symbolic entropy				– .12	– .27
Sum-normalized CSD					.49

## Discussion

In this paper, we presented the multiSyncPy package for computing multiple multivariate synchrony metrics. Our work aims to make such methods accessible while providing a good balance of alternatives, which the simple code examples presented earlier attempted to demonstrate. Relative to other software packages in the area of synchrony, multiSyncPy provides a valuable new contribution by focusing on multivariate synchrony, with applications in diverse areas of inquiry in the cognitive and behavioral sciences as well as other disciplines that might be interested in such phenomena (e.g., ecology, human–computer interaction).

Another contribution of our work is to present an initial investigation of how different metrics perform on the same tasks. The methods collated in multiSyncPy have previously only been introduced in isolation from one another, and this initial comparison of multivariate metrics is novel. By examining two types of synthetic data, we observed that some metrics are more responsive than others to the addition of correlated noise to a multivariate signal, and some metrics appear more sensitive to the coupling strength in situations that can be modeled as coupled Kuramoto oscillators. The ‘rho’ metric, for example, seemed the least influenced by increased correlated noise, while being one of the metrics that increased most quickly with the Kuramoto coupling strength parameter.

The synthetic data used in this paper are of course based on simplified mathematical models. They are useful, though, because they give the opportunity to change parameters of the data generation process and then observe corresponding trends in the values of synchrony metrics; however, there are some limitations worth noting. In particular, some of the interesting and complex properties of real-life signals may not be present in the data. One property that might be worthwhile to investigate in future work is quasi-periodicity, which is not reflected in our Kuramoto model since it is composed of oscillators following simple sinusoidal patterns. Methods for generating synthetic quasi-periodic data would make it possible to compare how different metrics perform under a wider range of conditions. Moreover, it is likely that a wide variety of nonlinear relationships between variables in a system are possible, and can be modeled in synthetic data. Future work could examine whether and how various nonlinear relationships impact synchrony metrics in different ways.

Overall, on data from real-life experiments, our metrics showed limited increases over a surrogate baseline when considering windows of data, although a significant effect was observed for four out of five metrics on the full ELEA recordings. The fact that the increases were frequently quite small or not statistically significant might indicate that it may be hard to detect group-level synchrony in team tasks, it may be less common than the more frequently-investigated phenomenon of dyadic synchrony in teams, or it could be that this form of surrogation is quite conservative compared to simple randomization (Moulder et al., 2018). Looking at our results on the data analyzed by Dale and colleagues (Dale et al., 2020), it seems that the dyadic synchronization studied by the original authors was more noticeable against a surrogate baseline. This is to say that it is important to consider that pairs within larger teams may move in and out of coordination with each other over time. Future work could more systematically investigate not only in more detail the relationship between the multivariate metrics (see Schoenherr et al., 2019 for inspiration), but also convergence in pair-wise versus group-level coordination metrics, and changes over time in multivariate coordination (Amon et al., 2019). We expect that a key differentiator is in measuring synchrony as a system-level construct, which may not be the same as an aggregation of the synchrony between component dyads. Since the change above the surrogate baseline was generally small in our case studies on real data, future work could also search for examples of situations where multivariate synchrony is more obviously present. And, more generally, much remains to be known about the conditions under which system-level synchrony emerges in the variety of domains we mentioned involving social interactions.

In terms of the practical utilization of our package, while these methods might now be more accessible than previously, we cannot understate the importance of carefully considering the methods, revisiting the original sources for these metrics, carefully inspecting the data to ensure the analyses are appropriate (e.g., periodic vs. non-rhythmic), choosing the correct pre-processing techniques (e.g., appropriate filtering, phase extraction, window size, time-norma etc.), analyzing multivariate systems with the same number of variables, and determining which segments of the data to analyze (removing transients, selecting for periods of activity vs. inactivity). As the field shifts from primarily bivariate to multivariate coordination dynamics, careful thinking, experimentation, and systematic comparison and validation of the multiple possible methods (including those presented in this paper and others such as (Zhang et al., 2020) and (Baboukani et al., 2019)) are required to fully understand these metrics.

In conclusion, multiSyncPy provides a range of synchrony metrics that can be computed easily through simple function calls in Python. These metrics come from a range of theoretical backgrounds, and the context may make some metrics more appropriate or informative than others. All of the metrics apply to multivariate time series, and so can be used to investigate system-level constructs of synchrony. System-level synchrony is under-researched, even in contexts where synchrony has been studied previously, such as small group interactions. Our methods are also appropriate in situations where there are numerous variables and it would be difficult to make sense of a large number of pairwise synchronizations. In other words, we aim to contribute tools that may advance the field's understanding of how coordination functions across scales (Kelso, 2021; Zhang et al., 2019). For the benefit of future researchers interested in multivariate coordination dynamics, multiSyncPy is made freely available under the liberal LGPL license.

## Figures and Tables

**Fig. 12:**
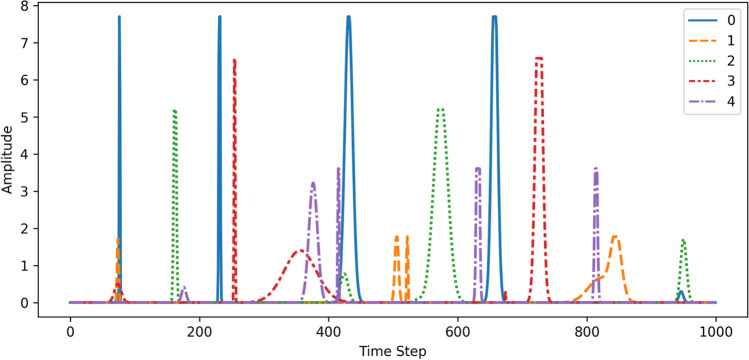
Example of synthetic data characterized by short bursts

**Fig. 13:**
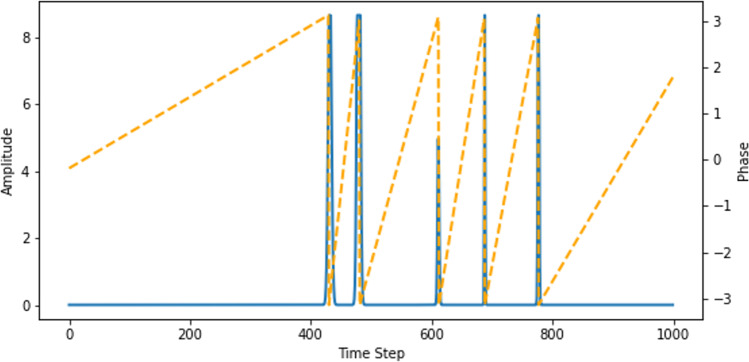
Example of synthetic data (*blue*) and corresponding extracted phase (*orange* and *dashed line*)

## Appendix I: Investigation of data characteristics on Kuramoto order parameter

This subsection provides supplementary results concerning how the test of the Kuramoto order parameter is affected by characteristics of the data, in particular comparing results from Kuramoto oscillators to signals consisting of short bursts or peaks of activity in an otherwise flat progression (such as the movement data we extracted from the ELEA corpus). Recall that, when describing the empirical case study on the ELEA movement data, the test on the Kuramoto order parameter may have provided misleading results on the data because it was characterized by short bursts of activity. To confirm that this is the case, we generated synthetic data with the relevant characteristics, and compared this to results from data generated by a Kuramoto model, where the same problem was not expected to occur.

First, we considered the unproblematic case using data from a Kuramoto model. Synthetic data generated by a Kuramoto model is moderated by the coupling strength, which is provided as a parameter. If the coupling strength is specified to be zero, then the components do not interact with each other and there is no mechanism by which they can coordinate and synchronize. In this situation, there is no synchrony expected. We generated a sample of 100 time series of five variables and 1000 time steps each from Kuramoto models where the coupling strength is 0. Performing the test of the Kuramoto order parameter, we find that, as expected, the result is not significant, *t*(99) = 0.25, *p* > .05. By contrast, we would expect significant levels of synchronization when using a higher value of coupling. To verify this, we generated a sample of 100 time series of five variables and 1000 time steps each where the coupling strength is 0.5. The result of the test in this instance is significant, *t*(99) = 7.35, *p* < .001. This gives some reassurance that the test works as expected when using signals that follow essentially sinusoidal progressions.

Next, we considered what happens when applying the test to data characterized by short bursts. False identification of significance might occur with such data in situations where no synchrony is present. We constructed relevant synthetic data by generating variables which exhibit short bursts of activity, but where the bursts have random location, amplitude, and duration. Each variable takes the form of a signal with a duration of 1000 time steps in which five bursts occur. Each burst is constructed from a Gaussian curve. The standard deviation of the curve is selected from an exponential probability distribution, and the amplitude of the curve is modified by multiplying by a number which is also randomly chosen from an exponential distribution. Finally, the curve is given a location which is selected from a uniform distribution over the 1000 time steps of the signal. As stated, five curves (or ‘bursts’) are added to a signal which has a value of 0 at all other time steps. Gathering multiple such variables together leads to time series in which no synchrony is expected. The parameters for the exponential distributions were chosen using manual inspection, so that the bursts in different synthetic signals would be narrow enough in duration to have limited coincidental overlap. Examples of the data generated using this method are shown in Fig. 12.

We generated a sample of 100 time series containing five variables each and applied the test on the Kuramoto order parameter. We found that although above chance-level synchrony was not expected, the results did suggest a significant degree of synchronization, *t*(99) = 62.6, *p* < .001. This confirms, using synthetic data generated especially for the purpose, that data characterized by short bursts is inappropriate for analysis with the Kuramoto test. This conclusion supports the remarks made in the “Empirical Data Results” subsection when interpreting the ELEA movement data, which are also characterized by short bursts. The explanation we propose is that the test assumes a uniform distribution of phase values, whereas there is no progression in phase during lengthy periods of inactivity, leading to a skewed distribution in data where activity occurs in short bursts. If the variables in a multivariate time series have skewed distributions of phase values, then the distribution of the average phase value at each instant (averaged across variables, not time) could be quite different from what is assumed by the test of the Kuramoto order parameter.

If the distribution of phase values is the source of the problem, it may be interesting to consider alternative phase extraction methods to the Hilbert transform used throughout this paper. Therefore, we tried again using two alternative methods. First, we used the continuous wavelet transform with the Morlet wavelet to extract the phase. Second, we took an approach similar to Gouhier and Guichard’s (2014) approach, in which we first find peaks (in our case using a method based on wavelet analysis) which are considered moments of maximal phase and then interpolate the phase linearly between these points.

The approach based on the Morlet wavelet brings about results which do not indicate significance, as is desired for the synthetic data, *t*(99) = 0.47, *p* > .05. However, further investigation shows that the problem is not entirely solved. When the synthetic data consists of bursts with longer duration, such that they can overlap more, then the test of the Kuramoto order parameter indicates significance, *t*(99) = 3.9, *p* < .001. Moreover, the test also indicates significance when applied to a surrogate baseline constructed from shuffling the variables between time series within the ELEA movement data. Using a surrogate baseline constructed from ELEA meetings with four participants, *t*(19) = 8.2, *p* < .001. This suggests that there are still issues when extracting phase using the continuous wavelet transform with the Morlet wavelet.

The approach based on peak-finding gives more reasonable values for the *t*-statistic compared to the results when the Hilbert transform is used, although significance is still concluded in the synthetic data, *t*(99) = 2.3, *p* < .05. Interestingly, when using the peak-picking approach, significance is not concluded from ELEA surrogate data, *t*(19) = 1.3, *p* > .05. One thing to note is that the outputs seem to be highly sensitive to changes in the parameters used by the peak-picking algorithm. We used visual inspection to identify reasonable values for the parameters. An example of a synthetic time series and its corresponding phase time series, as extracted through the peak-picking method, is displayed in Fig. 13.

## Declarations

### Availability of data and materials

The ELEA corpus is available by request from the Idiap Research Institute, https://www.idiap.ch/dataset/elea. The triadic body synchrony data used by Dale et al. (2020) is openly available at https://github.com/racdale/triadic-bodily-synchrony. The synthetic data used in this research can be generated using functions included in the multiSyncPy package.

### Code availability

The multiSyncPy package is openly available under the LGPL license. The active repository is https://github.com/cslab-hub/multiSyncPy, while the specific version used in this research is stored at https://osf.io/g6da7/. The package is also available on the Python Package Index (PyPI) and can be installed with pip. A Jupyter notebook for computing the synthetic data results is also available at https://osf.io/g6da7/. The OpenPose software used to pre-process the ELEA video recordings is available at https://github.com/CMU-Perceptual-Computing-Lab/openpose.
